# Synergistic autoinhibition and activation mechanisms control kinesin-1 motor activity

**DOI:** 10.1016/j.celrep.2022.110900

**Published:** 2022-05-31

**Authors:** Kyoko Chiba, Kassandra M. Ori-McKenney, Shinsuke Niwa, Richard J. McKenney

**Affiliations:** 1 Department of Molecular and Cellular Biology, University of California, Davis, 145 Briggs Hall, Davis, CA 95616, USA; 2 Frontier Research Institute for Interdisciplinary Sciences (FRIS), Tohoku University, Miyagi, 6-3 Aramaki Aoba, Aoba-ku, Sendai 980-0845, Japan; 3 Lead contact

## Abstract

Kinesin-1 activity is regulated by autoinhibition. Intramolecular interactions within the kinesin heavy chain (KHC) are proposed to be one facet of motor regulation. The KHC also binds to the kinesin light chain (KLC), which has been implicated in both autoinhibition and activation of the motor. We show that the KLC inhibits the kinesin-microtubule interaction independently from the proposed intramolecular interaction within KHC. Cargo-adaptor proteins that bind the KLC stimulated processive movement, but the landing rate of activated kinesin complexes remained low. Microtubule-associated protein 7 (MAP7) enhanced motility by increasing the landing rate and run length of the activated kinesin motors. Our results support a model whereby the motor activity of the kinesin is regulated by synergistic inhibition mechanisms and that cargo-adaptor binding to the KLC releases both mechanisms. However, a non-motor MAP is required for robust microtubule association of the activated motor. Thus, human kinesin is regulated by synergistic autoinhibition and activation mechanisms.

## In brief

Kinesin-1 (KIF5) transports cargo along microtubules. The mechanism of KIF5 movement is understood, but how the motor is autoinhibited and activated remains unclear. Chiba et al. report both synergistic autoinhibition and activation of the KIF5 tetramer and suggest that predominant models in the field for these processes need revision.

## Graphical abstract

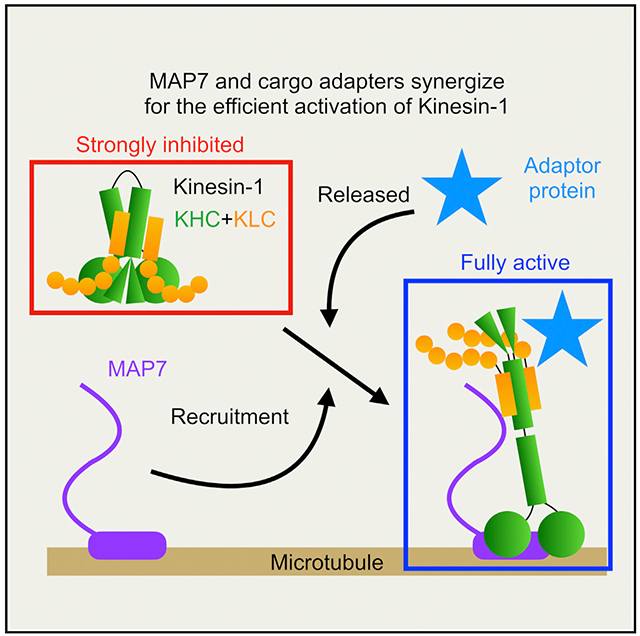

## INTRODUCTION

Microtubule (MT) motor proteins play important roles in the intracellular transport of many types of cargos (Hirokawa et al., 2009; Vale, 2003). Autoinhibition is a common mechanism to restrict the activity of the motors to prevent aberrant movement of cellular cargos. Recent studies have revealed how the minus-end-directed cytoplasmic dynein assumes an autoinhibited conformation that is relieved by cargo adapter molecules and its cofactor, dynactin (Reck-Peterson et al., 2018). In contrast, while kinesin-1 (“kinesin” hereafter) is the most ubiquitous plus-end-directed motor in cells, we currently lack a similarly detailed model of how kinesin is regulated. While prior studies have found that the kinesin motor is also regulated by autoinhibition (Dietrich et al., 2008; Friedman and Vale, 1999; Kaan et al., 2011; Verhey et al., 1998), our understanding of the mechanisms underlying kinesin inhibition and activation now lags behind that of dynein.

Kinesin predominantly exists as a heterotetrameric complex composed of a dimer of kinesin heavy chains (KHCs) bound to two copies of the kinesin light chain (KLC) subunit (Hirokawa et al., 1989; Vale et al., 1985). The N-terminal motor domain of kinesin utilizes ATP hydrolysis to produce force along MTs. The motor domain is followed by the coiled-coil stalk domain and a disordered C-terminal tail domain (Seeger et al., 2012). The KLC binds to the KHC stalk (Diefenbach et al., 1998; Verhey et al., 1998) via its N-terminal coiled coil (Verhey et al., 1998) and links various cargos to the KHC through its C-terminal tetratricopeptide repeat (TPR) domain (Pernigo et al., 2013).

In humans, three KHC (KIF5A–C) and four KLC (KLC1–4) genes comprise differentially expressed, biochemically distinct isotypes of kinesin heterotetramers. The isotypes show distinct expression patterns and produce unique phenotypes in mutant mice (Kanai et al., 2000; Rahman et al., 1998). Mutations in the neuronal isoforms KIF5A and KIF5C result in human neurodevelopmental or neurodegenerative pathologies (Crimella et al., 2012; Nicolas et al., 2018; Poirier et al., 2013; Reid et al., 2002; Willemsen et al., 2014). While these observations suggest functional differences exist among KIF5 isotypes, previous studies have analyzed KHCs from various species, and the relative biophysical properties of different kinesin isotypes from the same species have not been systematically compared under identical assay conditions.

Several studies have proposed a model whereby the motility of the KHC is regulated via direct interaction between the tail domain and the motor domain (Coy et al., 1999; Dietrich et al., 2008; Friedman and Vale, 1999; Kaan et al., 2011; Stock et al., 1999). It postulates that kinesin flexes at a “hinge” in the middle of the coiled-coil stalk, enabling a short, charged peptide within the tail, called the isoleucine-alanine-lysine (IAK) motif, to associate with the motor domains and inhibit enzymatic and MT-binding activities (Hackney and Stock, 2000; Kaan et al., 2011). This interaction is believed to restrict the movement of the motor domains, preventing movement along MTs (Cai et al., 2007).

The KLC is also involved in kinesin autoinhibition. The presence of KLC strongly decreases MT binding and motility of the KHC (Friedman and Vale, 1999; Verhey et al., 1998). It remains unclear whether the KLC strengthens the tail inhibition or independently regulates KHC activity. The autoinhibited kinesin is proposed to be activated by the binding of distinct adaptor proteins to either the KHC, KLC, or both (Blasius et al., 2007; Fu and Holzbaur, 2013; Sun et al., 2011; Twelvetrees et al., 2016, 2019; Verhey et al., 2001). However, these experiments were performed in cell lysates, and thus, it remains unclear whether adaptor proteins directly activate kinesin in the absence of other cellular factors.

Here, we studied the autoinhibition and activation of kinesin motor complexes from all human KHC isotypes. Surprisingly, widely utilized mutants that are thought to abolish the IAK-mediated inhibition within the KHC are still strongly repressed by KLC, suggesting that the mechanism of KLC inhibition is independent of the KHC tail-inhibition mechanism. We further show that the autoinhibited kinesin is partially activated by the W-acidic motif protein nesprin-4. However, nesprin-4 binding only mildly stimulates the motor’s landing rate onto MTs. MT association of the nesprin-activated motor complex is greatly enhanced by the inclusion of microtubule-associated protein 7 (MAP7 (Monroy et al., 2018; Sung et al., 2008). Our results reveal that neither cargo-adapter binding nor recruitment by MAP7 is sufficient to fully activate the kinesin heterotetramer. Instead, a synergistic activation mechanism composed of adapter binding to the KLC, coupled with specific recruitment to the MT by MAP7, is utilized by human kinesin motors. These results parallel that of the dynein activation mechanism by cargo adapters and the scaffolding MAP dynactin (McKenney et al., 2014, 2016; Schlager et al., 2014), suggesting that cargo-adapter-mediated release of autoinhibition, coupled with MAP recruitment to MTs, is a generalized mechanism for the regulation of long-distance transport by mammalian MT motor proteins.

## RESULTS

### Direct comparison of human KIF5 isotypes

We first compared the motor activity of all three human isotypes of the KHC (KIF5) using a tail-truncated K420 construct (Shimizu et al., 2000; Figures S1A and S1B). Because this truncation boundary contains only the minimum amount of the stalk domain required for dimerization, these constructs should report on the biophysical properties of the unregulated kinesin motor domain. The motor domains of KIF5A, B, and C are around 70% identical, suggesting that the core motor mechanism is highly conserved between human isotypes. Indeed, we observed similar behavior of all three isotypes in our single-molecule assay, where motors landed on MTs and often moved processively along them (Figures S1C–S1G). The landing rates of the truncated motors were similar between all isotypes (Figure S1D). Interestingly, the velocity of KIF5A (1,048 ± 173 nm/s, SD) was significantly faster than that of KIF5B or KIF5C (870 ± 147 and 887 ± 152 nm/s, SD, respectively; Figure S1E; Table 1), implying the mechanochemistry of KIF5A may differ from the other human KIF5 isotypes. Among all isotypes, a similar fraction of motors showed processive movement on MTs (Figure S1F), and the measured run lengths were not substantially different (Figure S1G; Table 1). Thus, under identical assay conditions, the three human KIF5 motors have similar biophysical properties except for the measurably faster velocity of KIF5A.

### Isotype-specific autoinhibition of kinesin dimers

**Figure 1.**
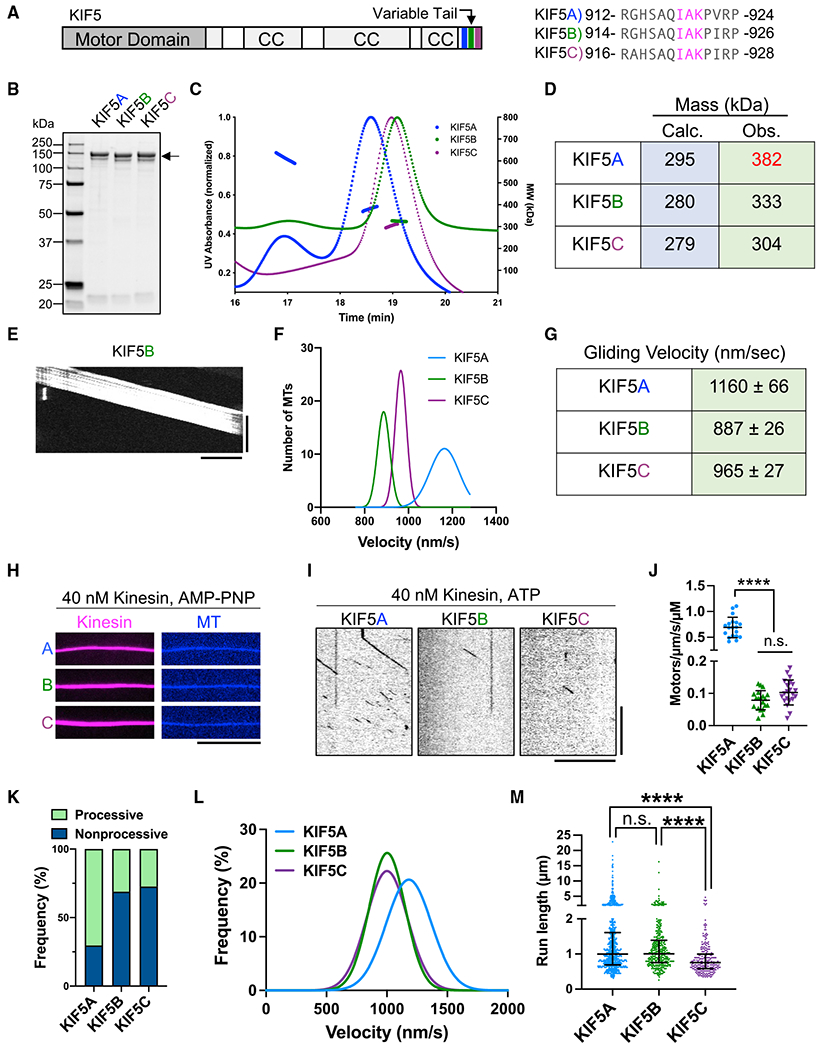
Characterization of full-length human KIF5 isotypes (A) Schematic of full-length KIF5 and alignment of the conserved IAK motif (magenta). CC, coiled coil. (B) Coomassie-blue-stained gel showing purified KIF5s (arrow). (C) Chromatogram and MALS fitting of KIF5A (blue), KIF5B (green), and KIF5C (purple). Normalized UV absorbance (dotted lines) and molar masses are plotted. (D) Table summarizing the calculated and the observed masses for each motor. (E) Kymograph of MT gliding with KIF5B. Scale bars: 20 s (vertical) and 5 μm (horizontal). (F) Gaussian fits of the KIF5 velocities from the gliding assay: 1,160 ± 66 (KIF5A), 887 ± 26 (KIF5B), and 965 ± 27 (KIF5C) nm/s; mean ± SD. N = 2; n = 90, 70, and 90 MTs. (G) Table summarizing the average gliding velocities of KIF5A-C. (H) TIRF images of KIF5A–C motors (magenta) bound to MTs (blue) in the presence of AMP-PNP. Scale bar: 10 μm. (I) Kymographs of KIF5A–C motors moving along MTs in the presence of ATP. Scale bars: 10 s (vertical) and 10 μm (horizontal). (J) Landing rates of KIF5A–C. Lines show mean ± SD: 0.69 ± 0.20 (KIF5A), 0.08 ± 0.03 (KIF5B), and 0.10 ± 0.04 (KIF5C)/μm/s/μM. N = 2; n = 19, 20, and 21 MTs. One-way ANOVA followed by Tukey’s multiple comparison test. ****adjusted p < 0.0001; ns, not significant. (K) Frequency of processive and non-processive (static or diffusive) events. Processive runs: 70%(KIF5A), 31% (KIF5B), and 27% (KIF5C). N = 2; n = 1,165, 1,209, and 901 molecules. (L) Gaussian fits of KIF5 velocities from single-molecule assay: 1,182 ± 187 (KIF5A), 1,002 ± 149 (KIF5B), and 1,001 ± 170 (KIF5C) nm/s; mean ± SD. N = 2; n = 820, 376, and 247 molecules. (M) Scatterplots showing the run length of KIF5A–C. Lines show median with quartile: 1.00 (0.69–1.61) μm (KIF5A), 1.00 (0.76–1.39) μm (KIF5B), and 0.76 (0.59–1.00) μm (KIF5C). Kruskal-Wallis test followed by Dunn’s multiple comparison test. ****adjusted p < 0.0001. See also Figure S2C and Table 1.

Because the largest difference in primary sequence between human KIF5 isotypes lies within the distal C-terminal tail domains (<20% identity in the tail region; Figure 1A), we purified recombinant full-length KHC isotypes (Figure 1B). We first characterized the oligomeric state of the purified proteins using size exclusion coupled with multi-angled light scattering (SEC-MALS). We found that KIF5B and KIF5C exist predominantly as dimers, as their measured molecular mass was within ~20% of the predicted dimeric value for each (Figures 1C and 1D). However, KIF5A deviated from its predicted dimeric molecular mass by greater than 20%, suggesting that a larger proportion of KIF5A molecules may exist in a more complicated oligomeric state (Figures 1C and 1D). Alternatively, it is possible that KIF5A is not amenable to MALS analysis or that the molecules are posttranslationally modified during expression, increasing their apparent molecular weight. Our KIF5A preparation also contained a smaller population that eluted earlier with a measured mass of ~611 kDa, close to that predicted for a tetramer of KIF5A heavy chains (590 kDa; Figure 1D). We conclude that full-length KIF5A has a higher propensity to oligomerize *in vitro* than that of KIF5B or KIF5C.

We then tested the motor activities of our kinesin preparations using a multi-motor MT-gliding assay in which the motors are attached nonspecifically to a glass surface. For all isotypes, we observed continuous movement of MTs across the glass surface (Figure 1E), confirming that all three recombinant motors were active. The MT gliding velocities of KIF5B and KIF5C were comparable, but we again observed a faster velocity of KIF5A than the other isotypes (Figures 1F and 1G). These results show that recombinant KIF5 dimers are active motors with similar biochemical and biophysical parameters as truncated motors.

Next, we studied the single-molecule behavior of human KHC isotypes using total internal reflection fluorescence (TIRF) microscopy. As all three human KHC isotypes contain the conserved “IAK” motif located within their tail domains (Figure 1A), based on prior findings (Dietrich et al., 2008; Kaan et al., 2011), we expected the MT-binding and motor activities of the full-length motors to be strongly attenuated as compared with the tail-truncated constructs (Figure S1). We first confirmed that all three isotypes bound to MTs in the presence of the ATP analogue, 5′-adenylylimidodiphosphate (AMP-PNP) (Figure 1H), in agreement with prior findings in cell lysates (Vale et al., 1985; Verhey et al., 1998). In the presence of ATP, we observed very few binding events along MTs at concentrations 100-fold higher than those used for the tail-truncated constructs (Figures 1I and 1J, compare with Figures S1C and S1D; Table 1). Quantification of the MT landing rate revealed KIF5A had a distinctively higher landing rate than KIF5B and KIF5C (Figure 1J; Table 1) and a much higher fraction of processive motors (Figure 1K). However, the landing rates of the full-length motors were several hundred-fold lower than those for the tail-truncated constructs (Figure S1D; Table 1), confirming that the presence of the tail domain strongly impacts the MT association rate of the motor.

Close inspection of the kymographs revealed that motile molecules of KIF5A were sometimes brighter than those of the other isotypes (Figure 1I), consistent with our findings that our preparation of KIF5A contains a fraction of oligomers (Figures 1C and 1D). The brighter KIF5A molecules were 2 ± 0.65 (SD)-fold brighter (n = 206 molecules; N = 2 trials) than the dimmer KIF5A molecules within the same chamber. We conclude that the brighter KIF5A molecules represent tetrameric motors, in support of our SEC-MALS data (Figures 1C and 1D). Consistently, the brighter KIF5A molecules often traveled longer distances than the dimmer KIF5A molecules or compared with KIF5B or KIF5C (Figures S2A, S2I, and S2M). We measured the brightness distribution of processive kinesin molecules for each KIF5 isotype and found that the intensity of KIF5A molecules was shifted toward higher values, consistent with a fraction of the molecules existing as oligomers (Figure S2B). Therefore, the higher landing rate (Figure 1J) and larger fraction of processive KIF5A molecules (Figure 1K) may result from oligomerization of the KIF5A motor in our assay.

We again observed uniquely fast behavior for KIF5A (Figure 1L), consistent with the truncated motors (Figure S1E; Table 1). Quantification of motor run lengths (1.00, 1.00, and 0.76 μm [median values] for KIF5A, B, and C, respectively) revealed shorter excursions for KIF5C (Figure 1M; Table 1), possibly revealing further distinctions within the KIF5 isotypes. These results confirm that the tail domain of kinesin inhibits its processive movement, consistent with prior findings (Friedman and Vale, 1999). We observe that the efficiency of tail-mediated inhibition varies between human KHC isotypes. Our results also reveal that the KIF5A dimer is more prone to form oligomers in our conditions, which we did not observe for KIF5B or KIF5C. This property may affect the tail-mediated inhibition of KIF5A. Finally, we cannot rule out the possibility that the C-terminal fluorophore may impact the autoinhibition mechanism of the motors, but note that it does prevent activation of the kinesin motor (see below).

### Association with the KLC further inhibits the kinesin-MT interaction

**Figure 2.**
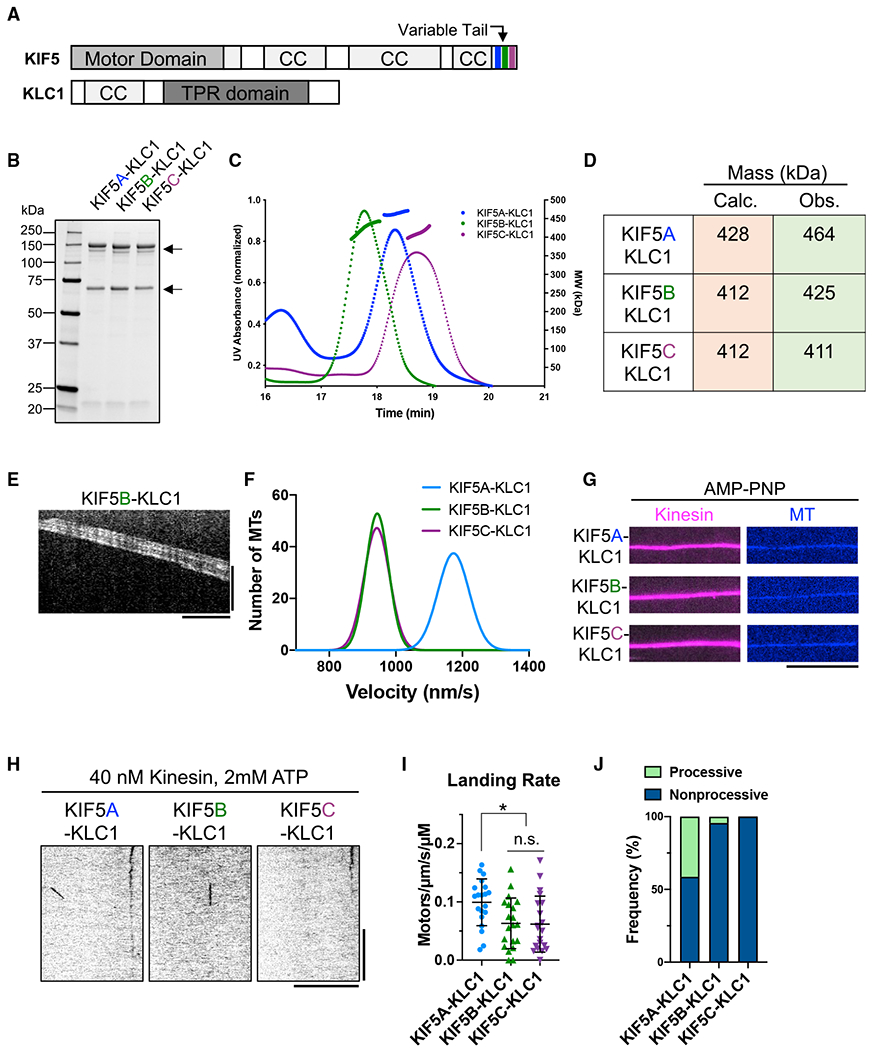
Characterization of the human KIF5-KLC heterotetramers (A) Schematic of full-length KIF5 and KLC1. (B) Coomassie-blue-stained gel showing purified heterotetramers (arrows). (C) Chromatogram and MALS fitting of KIF5A-KLC1 (blue), KIF5B-KLC1 (green), and KIF5C-KLC1 (purple). Normalized UV absorbance (dotted lines) and molar masses are plotted. (D) Table summarizing the calculated theoretical and the observed masses for each motor. (E) Kymograph of MT gliding with KIF5B-KLC1. Scale bars: 20 s (vertical) and 5 μm (horizontal). (F) Gaussian fits of the velocity histograms for KIF5A-KLC1 (blue), KIF5B-KLC1 (green), and KIF5C-KLC1 (purple) from the MT gliding assay: 1176 ± 47 (KIF5A-KLC1), 943 ± 33 (KIF5B-KLC1), and 944 ± 36 (KIF5C-KLC1) nm/s; mean ± SD. N = 2; n = 90 MTs, respectively. (G) TIRF images of KIF5-KLC1 (magenta) on MTs (blue) in the presence of AMP-PNP. Scale bar: 10 μm. (H) Kymographs of KIF5-KLC1 on MTs in the presence of ATP. Scale bars: 10 s (vertical) and 10 μm (horizontal). (I) Landing rates of KIF5-KLC1. Lines show mean ± SD: 0.10 ± 0.04 (KIF5A-KLC1), 0.06 ± 0.04 (KIF5B-KLC1), and 0.06 ± 0.05 (KIF5C-KLC1)/μm/s/μM. N = 2; n = 20 MTs, respectively. One-way ANOVA followed by Tukey’s multiple comparison test. *adjusted p < 0.05. (J) Frequency of processive and non-processive (static or diffusive) events. Processive runs: 41% (KIF5A-KLC1), 4% (KIF5B-KLC1), and 0% (KIF5C-KLC1). n = 290, 91, and 89 molecules. See also Table 1.

Previous studies have suggested that binding of the KLC to the KHC strongly represses the kinesin-MT association (Verhey et al., 1998) and enzymatic activity (Coy et al., 1999; Stock et al., 1999). We purified recombinant kinesin heterotetramers with all three human KHC isotypes co-expressed with the brain-specific KLC1 (Kawano et al., 2012; Rahman et al., 1998; Verhey et al., 1998) using a multi-gene baculovirus (Figures 2A and 2B). We measured a stoichiometric ratio of ~1.0:0.8 for KHC:KLC1 by quantitative Coomassie blue staining, and SEC-MALS analysis of the purified complexes further revealed a homogeneous population of heterotetrameric motor complexes for all three KHC isotypes, which measured within 10% of the expected molecular weight (Figures 2C and 2D).

We then tested the activity of the recombinant kinesin heterotetramers in the multi-motor MT gliding assay. Similar to the KHC dimers (Figures 1E and 1F), all three isotypes powered the smooth continuous gliding of MTs (Figure 2E). The gliding velocities of KIF5B and KIF5C were nearly indistinguishable (Figures 2F and S3A) and very similar to the gliding velocities of KHC dimers containing these isotypes (Figure 1F). We observed a significantly faster velocity for KIF5A-KLC1 (Figures 2F and S3A), confirming the distinct mechanochemistry of KIF5A. The similar gliding velocities for dimeric and heterotetrameric kinesin complexes is consistent with a previous report for kinesin complexes isolated from brain (Hackney, 1991). Thus, the KLC subunit does not impact the mechanochemistry of the KHC in a multi-motor assay, in which the motors are likely forced into an active conformation by binding to the glass surface.

Next, we assessed the ability of the purified heterotetramers to bind to MTs in the presence of AMP-PNP. As expected (Vale et al., 1985; Verhey et al., 1998), the presence of the KLC did not inhibit the binding of the motors to MTs (Figure 2G). In the presence of ATP, we observed little interaction of the purified heterotetramers with MTs at concentrations 100-fold higher than those used for the truncated motors (Figure 2H, compare with Figure S1C). Indeed, the landing rates of all three heterotetrameric motors (Figure 2I) were several hundred-fold lower than that of tail-truncated motors (Figure S1D) and comparable to that of the kinesin dimers (Figure 1J; Table 1). However, we found that KIF5A-KLC1 had a 7-fold lower landing rate than the KIF5A dimer, revealing that KLC binding leads to a more repressed motor with similar MT-association kinetics to KIF5B and KIF5C dimers or heterotetramers. This effect may be due to an artificially higher landing rate caused by KIF5A oligomerization in the absence of the KLC, as reported above.

Although MT-association events were rare, we quantified the percentage of events that were processive versus static or diffusive and observed that KIF5A heterotetramers showed a larger fraction of processive motors compared with KIF5B or KIF5C heterotetramers (Figure 2J). In addition, the fraction of processive KIF5B and KIF5C motors observed was decreased by the KLC (Figure 2J). Thus, in agreement with prior studies in cell lysates (Verhey et al., 1998), association of the KHC with the KLC leads to largely repressed heterotetrameric motor complex regardless of the KHC isotype. Given the higher ratio of KIF5A-KLC1 complexes that moved processively, we were able to quantify enough processive events to derive velocity and run-length distributions, which were similar to that of the KIF5A dimer (Figures S3B, S3C, 1L, and 1M). We conclude that the distinct mechanochemical properties of KIF5A we observe in tail-truncated (Figure S1) or full-length KHC complexes (Figure 1) also extend to heterotetrameric kinesin motor complexes. However, we cannot rule out that motile KIF5A-KLC1 motors represent KHC molecules that dissociated from the KLC. The data reveal that KLC-mediated motor inhibition synergizes with the tail-mediated inhibition for all human KHC isotypes.

### KLC-mediated autoinhibition is independent of tail-mediated autoinhibition

**Figure 3.**
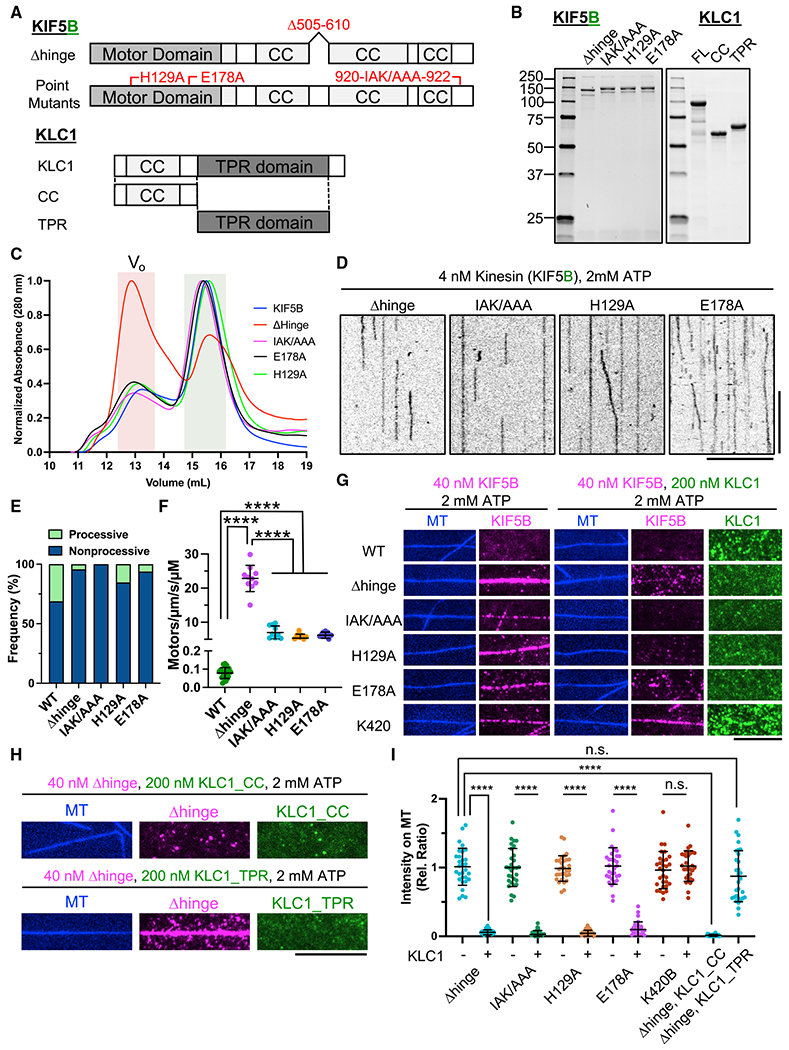
The KLC inhibits KIF5 independently of the tail-inhibition mechanism (A) Schematic of KIF5B-Δhinge, IAK/AAA, H129A, and E178A mutants and KLC1 and fragments used. KIF5B-Δhinge lacks amino acids (aas) 505–610. KLC1-CC or KLC1-TPR comprises aas 1–200 or aas 201–500 of KLC1. (B) Coomassie-blue-stained gel showing purified KIF5-mScarlet mutants and sfGFP-KLC1. (C) Normalized SEC chromatogram showing all KIF5B mutants and the wild-type (WT) protein. Red shade: void volume (V_o_) of the column; green shade: fractions of motors used for TIRF assays. (D) Kymographs of KIF5 mutants (in the absence of KLC) on MTs in the presence of ATP. Scale bars: 10 s (vertical) and 10 μm (horizontal). (E) Frequency of processive and non-processive (static or diffusive) events. Processive runs: 31% (WT), 4% (KIF5B-Δhinge), 0% (KIF5B-IAK/AAA), 15% (KIF5B-H129A), and 6% (KIF5B-E178A). N = 2; n = 20 MTs; n = 280, 325, 367, and 645 molecules. Data for KIF5B-WT are replotted from Figure 1K. (F) Landing rates of KIF5 mutants. Lines show mean ± SD: 0.08 ± 0.03 (KIF5B-WT; data are replotted from Figure 1J), 22.8 ± 3.8 (KIF5B-Δhinge), 7.0 ± 2.0 (KIF5B-IAK/AAA), 5.4 ± 1.2 (KIF5B-H129A), and 6.2 ± 0.9 (KIF5B-E178A)/μm/s/μM. N = 2; n = 20 MTs, respectively. One-way ANOVA followed by Tukey’s multiple comparison test. ****adjusted p < 0.0001. (G) TIRF images of 40 nM KIF5B (magenta) on MTs (blue) with or without 200 nM recombinant full-length KLC1 (green) in the presence of ATP. Scale bar: 10 μm. (H) TIRF images of 40 nM KIF5B-Δhinge (magenta) on MTs (blue) with 200 nM recombinant KLC1 (KLC1-CC or KLC1-TPR) in the presence of ATP. Scale bar: 10 μm. (I) Relative fluorescence intensity showing the effect of KLC1 on MT binding of KIF5B shown in G and H. Lines: mean ± SD. N = 2; n = 30 MTs. A two-tailed unpaired Student’s t test (KIF5B-IAK/AAA, KIF5B-H129A, KIF5B-E178A, and K420B in the presence or absence of KLC1) or one-way ANOVA followed by Tukey’s multiple comparison test (KIF5B-Δhinge alone and KIF5B-Δhinge with KLC1, KLC1_CC, and KLC1_TPR). ****p < 0.0001.

Previous studies on tail-mediated KHC autoinhibition utilized different types of mutations that are thought to bypass the molecular mechanism responsible for motor inhibition (Coy et al., 1999; Friedman and Vale, 1999; Kaan et al., 2011; Kelliher et al., 2018). First, deletion of the hinge region within the central stalk was shown to result in higher MT-stimulated ATPase activity (Coy et al., 1999; Friedman and Vale, 1999) and a variable increase in the number of processive molecules observed in a single-molecule assay (Friedman and Vale, 1999). The interpretation of these results was that, without the hinge region, the KHC could not fold back on itself to facilitate an interaction between the C-terminal IAK domain and the N-terminal motor domains. However, evidence of kinesin failing to adopt a folded state was lacking in these studies. Contradictory data showed an apparent increase in sedimentation value for delta-hinge kinesin constructs, indicating potential oligomerization of the mutated motor (Coy et al., 1999). An atomic structure of dimeric *Drosophila* kinesin motor domains bound to the IAK peptide revealed important conserved residues within the motor domain that interact with the tail domain (Figure 3A; Kaan et al., 2011). Mutation of these critical residues has been reported to disrupt kinesin autoinhibition in cells (Cai et al., 2007), enzymatic assays (Hackney and Stock, 2000), and in single-molecule measurements performed in cell lysates (Kelliher et al., 2018). In addition, the KLC has been reported to contribute to kinesin autoinhibition in a manner that is distinct from the IAK-mediated kinesin repression in cells (Cai et al., 2007).

We generated some of these mutations in KIF5B (Figures 3A and 3B) in order to examine their effects on motor activity under identical assay conditions. We also isolated KLC1, and constructs encompassing its N-terminal and C-terminal domains (Figures 3A and 3B), in order to directly observe how the KLC affects each type of autoinhibition mutant. We first analyzed these mutants using SEC and observed that the point mutants eluted indistinguishably from wild-type KIF5B (Figure 3C). The delta-hinge mutant showed a large population of presumably oligomerized motors that eluted in the void volume of the column but a second population that eluted very similarly to wild-type KIF5B and the point mutants. The large population of delta-hinge oligomers may explain the previously reported increase in sedimentation value during density gradient separation (Coy et al., 1999). We utilized the delta-hinge molecules that eluted similarly to wild-type kinesin (Figure 3C) in our further experiments. We conclude that mutations that are predicted to prevent the head-tail interaction do not result in large changes to the molecular shape of the motor as assessed by SEC. However, removal of the hinge region results in substantially more oligomerization or aggregation of the mutant motor.

In our single-molecule TIRF assay (Figure 3D), we observed very little processive movement of the mutant dimers (Figures 3D and 3E). These observations are in contrast to a previous report of an increased fraction of processive delta-hinge mutant motors in a single-molecule assay (Friedman and Vale, 1999), although the increase in processive motors was variable in that study. In addition, the delta-hinge mutant, as well as mutation of residue K944 within *Drosophila* kinesin IAK domain (equivalent to K922 in human KIF5B), reportedly increased the velocity and distance traveled between pauses of single kinesin molecules in cell lysates (Kelliher et al., 2018). These observations were interpreted as evidence for decreased efficiency of tail-mediated autoinhibition, although an increase in the fraction of processive motors was not reported. However, we did not observe an increase in the fraction of processive motors when we assessed two equivalent mutations (Δhinge and IAK→AAA) in human KIF5B (Figures 3D and 3E). Finally, mutation of residues within the *Drosophila* kinesin motor domain prevented tail-peptide-mediated inhibition of the motor’s enzymatic activity (Kaan et al., 2011), which was again interpreted as evidence for disruption of the tail-mediated inhibition mechanism. In contrast to these prior observations, we did not detect an increase in the fraction of processive mutant motors in our purified system using the equivalent mutations H129A and E178A in human KIF5B (Figures 3D and 3E). We instead observed a relative decrease in the fraction of processive motors and a consistently large, ~50- to 100-fold increase in the MT landing rate of all of the mutant motors assayed (Figure 3F), in agreement with previous notions that the IAK domain inhibits the MT association of the motor (Cai et al., 2007; Stock et al., 1999). From these data, we conclude that the main effect of abolishing the IAK-motor interaction is a more than 10-fold increase in the MT-association rate, but not a substantial increase in processive motility. It is unclear why the fraction of processive motors is reduced by these mutations (Figure 3E), but the magnitude of that effect is greatly exceeded by the much larger increase we observe in the MT-association rate. Our data therefore suggest that other elements within the mutant motors keep the motor in a conformation incompatible with processive movement. One candidate is the stalk region proximal to the motor domain, which was recently shown to modulate the kinesin-MT interaction (Hooikaas et al., 2019).

The KLC is reported to strongly inhibit the MT association of the KHC in cell lysates (Verhey et al., 1998) and in purified systems (Coy et al., 1999; Friedman and Vale, 1999). Fluorescence resonance energy transfer (FRET) measurements in cells suggested that the C-terminal TPR domain of the KLC was necessary to push the kinesin motor domains apart into a conformation that was incompatible with motility (Cai et al., 2007). For this to occur, the tail region of the motor is thought to fold at the hinge region to localize the KLC TPR domains near to the motor domains. To test this idea, we added purified KLC domains to KIF5B motors in *trans* and assessed the ability of the KHC to bind to MTs in the presence of ATP. As we previously observed, wild-type KIF5B had a low apparent affinity for MTs (Figures 3F and 3G), while in agreement with our landing-rate measurements (Figure 3F), the mutant KIF5B motors all showed substantially higher MT association (Figures 3F–3I) in the absence of the KLC. When a 5-fold molar excess of purified KLC was included, we observed strong attenuation of the MT association for all of the KHC motors tested (Figures 3G, 3I, S4A, and S4C). This result is not consistent with the current models for kinesin autoinhibition proposing that the tail must fold back to allow the IAK domain to bind to the motors and also to place the KLCs in close proximity to the motor domains (Cai et al., 2007; Coy et al., 1999; Dietrich et al., 2008; Friedman and Vale, 1999). In theory, this should not be possible when the hinge region is removed or when residues critical for the interaction between the tail and motor domains are altered. Purified KLC did not directly bind to MTs, and it did not affect the MT association of tail-truncated K420 (Figures 3G, 3I, and S4B), suggesting that the KLC does not block motors from binding MTs nonspecifically. Finally, in agreement with prior findings (Verhey et al., 1998), we found that the N-terminal domain, but not the C-terminal domain of the KLC, was sufficient to strongly attenuate the MT association of the delta-hinge KHC mutant (Figures 3H, 3I, and S4C). Thus, the N-terminal region of the KLC represses MT binding of the KHC, even in the absence of the proposed direct interaction between the tail and motor domains that underlies the predominant autoinhibition model in the field (Verhey et al., 2011). We conclude that the current model for the mechanism of kinesin autoinhibition is insufficient to account for these observations.

### Binding of a W-acidic motif cargo adapter to the KLC is sufficient to relieve kinesin heterotetramer autoinhibition

**Figure 4.**
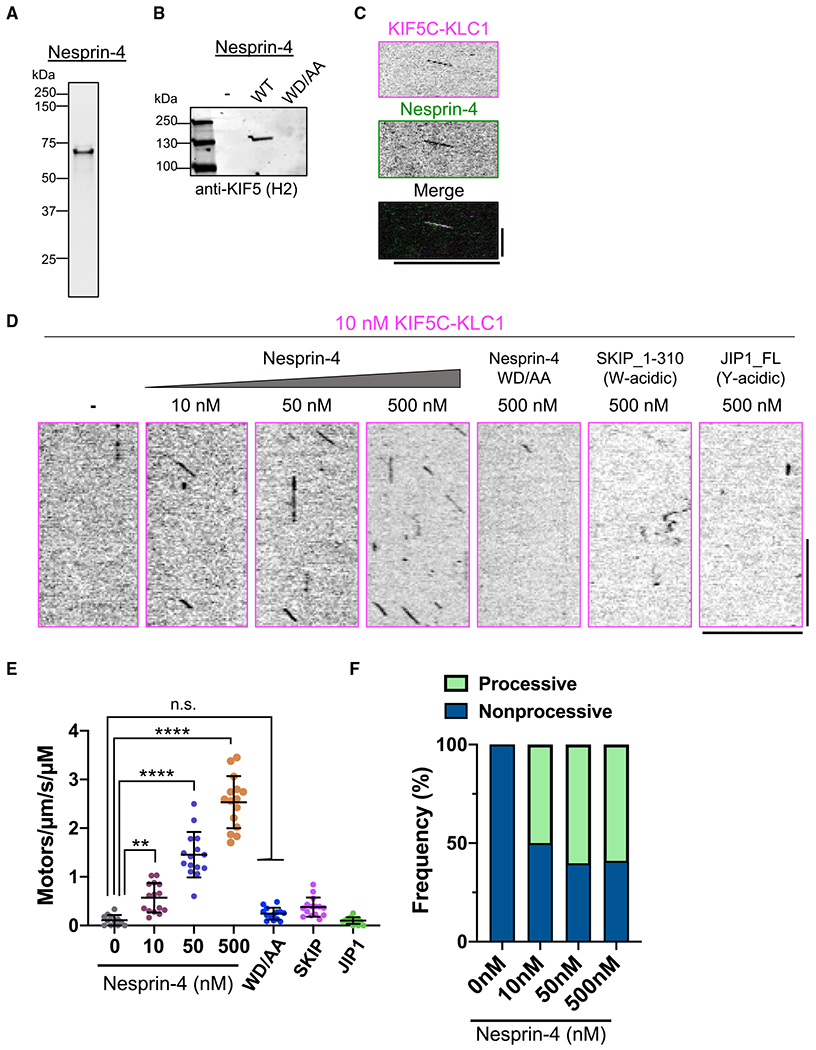
Nesprin-4 activates the autoinhibited KIF5-KLC heterotetramer (A) Coomassie-blue-stained gel showing purified sfGFP-tagged cytosolic domain of nesprin-4. (B) Immunoblot showing that KIF5 is coprecipitated with recombinant nesprin-4, but not its WD/AA mutant from brain lysate. (C) Kymograph showing co-movement of KIF5C-KLC1-mScarlet (magenta) with sfGFP-nesprin-4 (green) on MTs in the presence of ATP. Ten nanomolars of each protein was used. Individual fluorescent channels (top and middle) and the merged channel (bottom) are shown. Scale bars: 10 s (vertical) and 10 μm (horizontal). (D) Kymographs showing the motility of KIF5C-KLC1 mixed with indicated proteins in the presence of ATP. Scale bars: 10 s (vertical) and 10 μm (horizontal). (E) Landing rates of KIF5-KLC1. Lines show mean ± SD: 0.1 ± 0.1 (note, data for 10 nM KIF5C-KLC1 are replotted from Figure 2I), 0.6 ± 0.3 (10 nM KIF5C-KLC1 + 10 nM nesprin-4), 1.5 ± 0.5 (10 nM KIF5C-KLC1 + 50 nM nesprin-4), 2.5 ± 0.5 (10 nM KIF5C-KLC1 + 500 nM nesprin-4), 0.2 ± 0.1 (10 nM KIF5C-KLC1 + 500 nM nesprin-4_WDAA), 0.4 ± 0.2 (10 nM KIF5C-KLC1 + 500 nM SKIP_1–310), 0.1 ± 0.1 (10 nM KIF5C-KLC1 + 500 nM JIP1)/μm/s/μM. N = 2; n = 12, 15, 15, 15, 14, 15, and 15 MTs. One-way ANOVA followed by Dunnett’s multiple comparison test. **adjusted p < 0.01; ****adjusted p < 0.0001. (F) Frequency of processive and non-processive (static or diffusive) events. Processive runs: 0%, 24%, 50%, and 60% for 10 nM KIF5C-KLC1 with 0, 10, 50, and 500 nM nesprin-4, respectively. n = 89, 17, 112, and 221 molecules. Data for 0 nM nesprin-4 are replotted from Figure 2J.

Previous work delineated two classes of KLC-binding motifs that directly interact with the TPR domains of the KLC (Cross and Dodding, 2019). Attachment of the W-acidic sequence to intracellular cargo results in kinesin-dependent displacement of cargo in cells (Kawano et al., 2012). However, it remains undetermined whether binding of the W-acidic sequence to the KLC within the kinesin heterotetramer is sufficient to activate the autoinhibited motor or whether other cellular factors participate in motor activation. To examine whether the binding of W-acidic motifs activates the motor, we purified recombinant nesprin-4, a nuclear-membrane protein (Roux et al., 2009; Wilson and Holzbaur, 2015). Nesprin-4 contains a “LEWD” motif known to interact with the KLC. We confirmed that the cytoplasmic domain of nesprin-4 interacted with endogenous kinesin from rat brain lysates in a pull-down assay (Figures 4A and 4B). Importantly, mutation of the critical LEWD sequence to “LEAA” completely abolished kinesin binding, as expected (Figure 4B; Roux et al., 2009; Wilson and Holzbaur, 2015).

Next, we combined recombinant KIF5C-KLC1 heterotetramer with purified nesprin-4 in our single-molecule assay. W-acidic peptides have low micromolar affinities for isolated KLC TPR domains (Pernigo et al., 2013), and the native KLC is reportedly autoinhibited (Yip et al., 2016), making observation of single-molecule interactions at low nanomolar concentrations difficult. Nonetheless, at 10 nM concentration of each protein, we observed infrequent processive events that contained both KHC-mScarlet and sfGFP-nesprin-4 signals, confirming a direct interaction between the kinesin heterotetramer and nesprin-4 during processive motility (Figure 4C). We observed that 77% of processive kinesin molecules contained nesprin-4 signal, revealing that most motile kinesin complexes were bound to nesprin-4. Higher concentrations of nesprin-4 obscured the nesprin-4 signal due to high background, but we observed a clear dose-dependent increase in the frequency of kinesin landing (Figures 4D and 4E). Of these landing events, we observed a significant increase in the percentage of processive kinesin events (Figure 4F). In agreement with our pull-down data (Figure 4B), we did not observe any increase in landing events in the presence of the nesprin-4 LEAA mutant (Figures 4D–4F). The velocity and run lengths of the activated kinesin heterotetramers were similar across nesprin-4 concentrations (Figures S5A and S5B), suggesting the cargo-activated heterotetramer has similar biophysical properties to the uninhibited motors (Figures S1E and S1G). Finally, we confirmed that the addition of nesprin-4 also resulted in an increase of processive motors for the other KIF5 isotypes complexed with KLC1, revealing a conserved mechanism of activation for all human KIF5 isotypes (Figures S5C and S5D). Thus, in a purified system, the binding of a W-acidic motif adapter molecule to the kinesin heterotetramer is sufficient to relieve an autoinhibition mechanism that prevents processive movement along MTs.

We also tested an N-terminal fragment of the kinesin adapter protein SKIP, which contains tandem W-acidic motifs and binds to the isolated KLC TPR domain (Pernigo et al., 2013). Despite the well-characterized, albeit weak, binding of SKIP to the isolated KLC TPR domain, we did not observe any effect of this fragment in our single-molecule assay (Figures 4D and 4E). We additionally tested the Y-acidic motif adapter protein, JIP1, which binds directly to the KLC TPR domain (Pernigo et al., 2018) and is reported to activate KHC motility in cell lysates (Fu and Holzbaur, 2013). We again did not observe any effect of JIP1 on the frequency of processive events with our purified kinesin heterotetramer (Figures 4D and 4E). These results reveal that distinct adapters have differential abilities to activate the motor *in vitro.* We speculate that binding affinity, and potentially unexplored autoinhibition of the adapter molecules, likely play key roles in the activation process. Further work is needed to dissect the interactions of distinct adapter proteins with the heterotetrameric kinesin motor complex.

### MAP7 enhances kinesin heterotetramer activation

Despite the effect of nesprin-4 on the autoinhibited kinesin heterotetramer, we noticed that the landing rate of the activated kinesin-nesprin complexes (Figure 4E) was ~50-fold lower than that of the tail-truncated dimers (Figure S1G). This observation suggests that, while the inhibition of motor activity is relieved by the binding of an activating adapter, the full potential of the kinesin-MT interaction remains stifled. We reasoned that the low landing rate of activated kinesin motors could be due to the lack of a MAP7 family protein in our reconstitution. MAP7 has emerged as a powerful regulator of the kinesin-MT interaction *in vitro* and *in vivo. In vivo,* MAP7 acts as a critical cofactor for kinesin transport (Hooikaas et al., 2019; Pan et al., 2019; Serra-Marques et al., 2020; Tymanskyj et al., 2018), revealing its previously unknown importance in kinesin-based transport *in vivo. In vitro,* MAP7 family proteins greatly enhance the binding of kinesin to the MT lattice and have been proposed to activate the autoinhibited kinesin dimer (Ferro et al., 2022; Hooikaas et al., 2019; Monroy et al., 2018). The kinesin-binding domain of MAP7 can increase the landing rate of tail-truncated kinesin, suggesting that elements outside of the kinesin tail domain may also contribute to motor regulation (Hooikaas et al., 2019). However, the effects of MAP7 on the autoinhibition mechanism of the kinesin heterotetramer remain unexplored.

**Figure 5.**
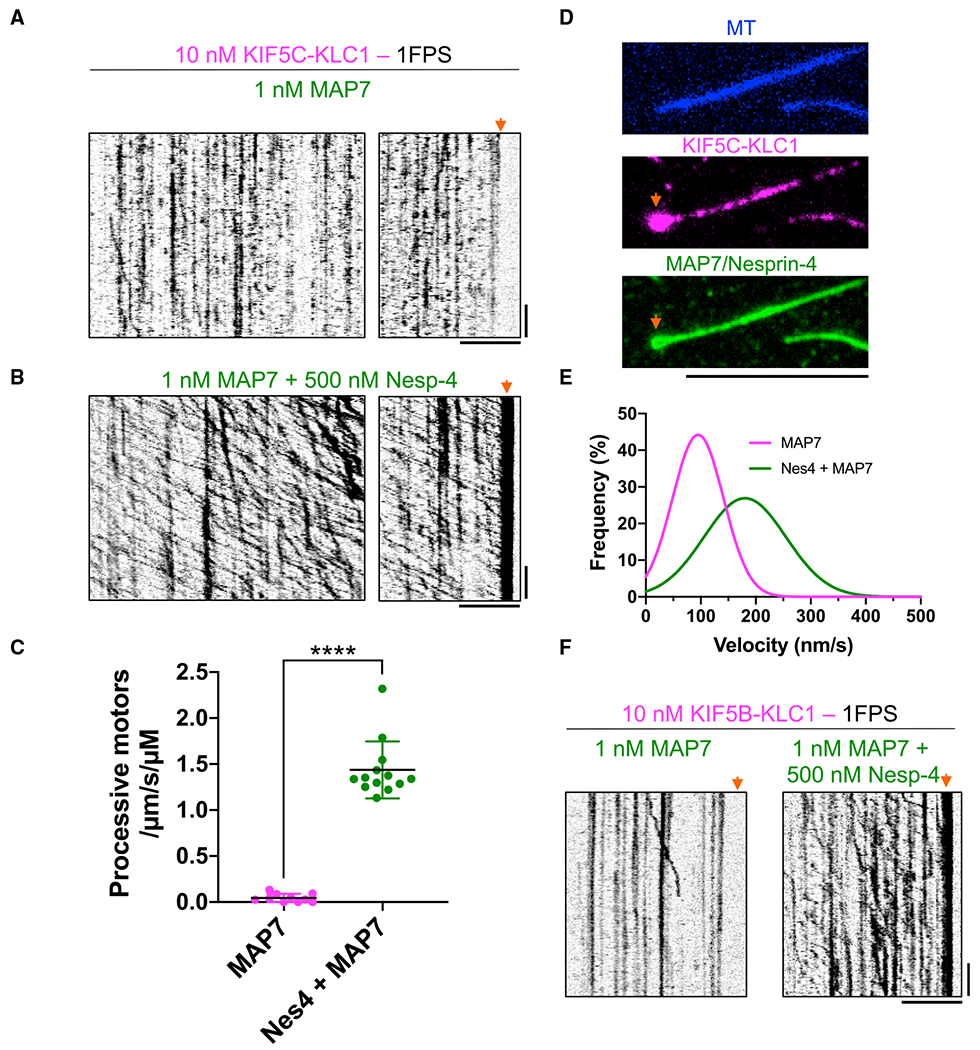
Synergistic activation of kinesin heterotetramers by nesprin-4 and MAP7 (A) Kymographs of KIF5C-KLC1 on MAP7-coated MTs. Note the lack of processive movement. Arrow highlights no accumulation of motors at the MT ends. Scale bars: 30 s (vertical) and 5 μm (horizontal). (B) Kymographs of KIF5C-KLC1 on MAP7-coated MTs in the presence of nesprin-4. Note the highly processive movement. Arrow highlights strong accumulation of processive motors at one end of the MT. Scale bars: 30 s (vertical) and 5 μm (horizontal). (C) Landing rates of processive KIF5C-KLC1 motors in the absence or presence of MAP7. Only processive molecules were counted in this analysis. Lines: mean ± SD: 0.045 ± 0.045 (KIF5C-KLC1 with MAP7) and 1.4 ± 0.31 (KIF5C-KLC1 with MAP7 and nesprin-4)/μm/s/μM. N = 2; n = 10 or 13 MTs. A two-tailed Student’s t test. ****p < 0.0001. (D) TIRF images of KIF5C-KLC1 (magenta, middle) accumulation at one MT end (arrows) in the presence of MAP7 and nesprin-4 (both green, bottom). Note nesprin-4 accumulation at the MT end is presumably nesprin-4 transported there by kinesin. Scale bar: 10 μm. (E) Gaussian fits of KIF5C-KLC1 velocities distributions in the presence of MAP7 or MAP7 + nesprin-4. Mean ± SD: 95 ± 47 nm/s (MAP7, magenta) and 180 ± 75 nm/s (MAP7 + nesprin-4, green). N = 2; n = 21 or 706 molecules. (F) Kymographs of KIF5B-KLC1 on MAP7-coated MTs with (right) or without (left) nesprin-4. Arrows indicate MT ends. Note accumulation of processive motors at MT ends only in the presence of MAP7 and nesprin-4. Scale bars: 30 s (vertical) and 5 μm (horizontal).

First, we examined the effects of MAP7 on the KIF5C-KLC1 heterotetramer and observed a strong increase in the number of motors binding to MTs (Figure 5A, compare with Figure 2H). Although the slower frame rates at which we acquired these data preclude a direct quantitative comparison with our data of the kinesin heterotetramer alone (Figure 2), the presence of MAP7 on the MT clearly increased the overall amount of the KIF5C-KLC1 on the MT. However, MAP7 did not obviously lead to the activation of processive motility, as we very rarely observed processive movement of the bound KIF5C-KLC1 heterotetramers (Figure 5A). Thus, we conclude that MAP7 strongly recruits the kinesin heterotetramer to MTs but does not relieve the autoinhibition of the complex in the absence of a cargo-adapter molecule.

Next, we added both nesprin-4 and MAP7 to the assay to assess the synergistic effects of nesprin-4-mediated release of autoinhibition and recruitment of motors by MAP7. Inclusion of both molecules in the assay resulted in a striking enhancement of processive kinesin heterotetramers along MTs (Figure 5B). The presence of MAP7 and nesprin-4 together stimulated the landing of processive kinesin motors by over 30-fold (Figure 5C), as compared with MAP7 alone. The motility of nesprin-4-activated KIF5C-KLC1 in the presence of MAP7 was distinct from the behavior of nesprin-4-activated motors alone (compare Figures 5B and 4D). In the presence of MAP7, activated motor movement was highly processive, with run lengths that clearly exceed 5 μm (Figure 5B). Such long runs were never observed in the absence of MAP7. Indeed, we noted a strong accumulation of motors at the presumed plus end of the MT (Figures 5B and 5D), suggesting that many motors moved until they reached the end of the filament. The weak affinity between nesprin-4 and kinesin precluded the dilution of motors in the assay, and the resulting high density of motors recruited to the MT by MAP7 made accurate assignment of the beginning and endings of runs difficult. We therefore measured uninterrupted segments of processive movements in the kymographs and found that, in the presence of MAP7, the run length of KIF5C-KLC1 was at least 3.5 μm, approximately 4-fold longer than we observed for nesprin-activated heterotetramers in the absence of MAP7 (Figure S5B). We note that this value is an underestimate of the true run length due to the limitations of motor concentration in the assay. Differences in image-acquisition rates precluded a direct quantitative comparison with the measured run lengths of nesprin-4-activated motors in the absence of MAP7 (Figure S5B), but the stimulation of kinesin heterotetramer processivity we observe in the presence of MAP7 is in agreement with a more modest increase reported for tail-truncated or full-length dimeric kinesin (Hooikaas et al., 2019; Monroy et al., 2018). We also measured the velocity of the rare processive molecules in the presence of MAP7 alone and the processive molecules observed in the presence of both MAP7 and nesprin-4. The velocity of processive kinesin in the presence of MAP7 was low (95 ± 46.5 nm/s and 180 ± 74 nm/s [mean ± SD] for MAP7 and MAP7 with nesprin-4, respectively; Figure 5E), also in agreement with prior work showing MAP7 hinders kinesin velocity (Ferro et al., 2022; Hooikaas et al., 2019; Monroy et al., 2018), although the magnitude of velocity decrease is larger in our assay. This velocity is well below that measured for kinesin-based cargo *in vivo,* and the molecular reason for it warrants further investigation. Recent structural data revealed that MAP7 and kinesin occupy partially overlapping binding sites on the MT, and thus, the density of MAP7 on the MT strongly affects kinesin’s velocity, suggesting the concentration of MAP7 in the reconstitution may be further tuned for optimal kinesin velocity (Ferro et al., 2022). Finally, we also observed the synergy of MAP7 and nesprin-4 with the more ubiquitously expressed KIF5B-KLC1 heterotetramer (Figure 5F), revealing a general pathway for kinesin activation through the binding of a cargo-adapter molecule to the light chains and recruitment of the activated complex to MTs via MAP7 family proteins.

## DISCUSSION

We have observed isotype-specific differences in the mechanochemistry of truncated, uninhibited motors, finding that KIF5A motors are distinctly faster among the three human isotypes. In the absence of the KLC, the KIF5A heavy chain has a higher tendency to oligomerize *in vitro* (Figures 1 and 2). This result may have implications for human disease, as mutations in the divergent KIF5A tail domain cause amyotrophic lateral sclerosis (ALS) (Nicolas et al., 2018), which may conceivably alter the oligomerization properties of the motor.

We find evidence that the degree of tail-mediated autoinhibition varies by KHC isotype. Despite conservation of the IAK motif in all KIF5 genes, tail-mediated repression of motor activity is weakest for KIF5A, which may be related to its divergent C-terminal extension and higher tendency to oligomerize. It is currently unclear what fraction of the total cellular pool of KHC operates in the absence of the KLC. Certain functions of kinesin motors may be independent of the KLC (Palacios and St Johnston, 2002), such as the transport of mitochondria (Glater et al., 2006; van Spronsen et al., 2013), although the data for this assertion are conflicting (Khodjakov et al., 1998). Thus, understanding of the roles of the divergent kinesin tail domains in the autoinhibition mechanism of the KHC dimer will inform on cargo-specific regulation of kinesin motor activity.

Consistent with prior work (Verhey et al., 1998), we find that association of the KLC with the KHC leads to a fully repressed motor that is incapable of productive interactions with the MT. While Verhey et al. suggested the distal tail region of KHC is required for the KLC-mediated regulation, our data show that the IAK domain is not required for KLC-mediated regulation. Binding of the KLC does not prevent motor association with MTs in the presence of an AMP-PNP, implying an allosteric mechanism, as opposed to steric occlusion of the motor domain. Interestingly, only the KLC N terminus is required for this effect (Verhey et al., 1998; Figure 3). Previous studies proposed a model whereby the kinesin molecule folds in half to allow direct interactions between the motor and tail domains (Verhey et al., 2011). A folded conformation would place the KLC adjacent to the motor domain, possibly facilitating direct steric effects. However, we found that removing the hinge region, or mutating residues important for this putative motor-tail interaction, did not prevent the KLC from exerting its inhibitory effect on the motor-MT interaction. The current intramolecular folding model cannot account for these data. Since the binding site for the KLC is separated from the motor domain by ~450 amino acids, we hypothesize that a long-range allosteric mechanism could explain this effect. Such a mechanism could arise from the propagation of conformational changes down the central coiled-coil stalk region of kinesin upon KLC association, perhaps through changes in the registry of the coiled coils, as has been observed in the dynein stalk domain and the dynein activator BicD2 (Carter et al., 2008; Cui et al., 2020; Kon et al., 2009; Liu et al., 2013).

Moreover, our data with KHC dimers are also not fully consistent with the current model for kinesin autoinhibition. Prior studies have suggested that removal of either the hinge region or the IAK domain leads to motor activation (Coy et al., 1999; Friedman and Vale, 1999; Kelliher et al., 2018). However, some of these studies did not directly assay motor movement as we have done here. Friedman and Vale reported an increased frequency of processive KHC dimers when the hinge region was deleted (Friedman and Vale, 1999). However, this effect varied from 5- to 50-fold across the two protein preparations reported. In addition, the motor velocity was 3-fold slower than that of the nesprin-4-activated motors reported here, possibly due to differing assay conditions. Kelliher et al. (2018) reported that deletion of the hinge region or mutation of residues important for motor domain-IAK interaction in *Drosophila* kinesin-1 led to an increase in motor velocity and run length between run pauses. These results were interpreted as evidence of disruption of KHC autoinhibition, but the study did not compare the landing frequency of motile motors. Our results with similar mutations in the human KHC suggest the primary effect is to increase the landing frequency of motors onto the MT. If a fraction of motors is inherently active, increasing the landing rate would result in more observed motile motors, even without a change in the proportion of active motors within the population. An increased landing rate could also account for the observed enhancement of kinesin enzymatic activity upon deletion of the hinge region, or motor-IAK interactions (Coy et al., 1999; Kaan et al., 2011), if the motors turn over ATP without coupling it to processive stepping. Consistent with this hypothesis, we observe that mutant KHC motors bind and release from the MT in the presence of ATP (Figure 3). Thus, our data revise the current models for KHC autoinhibition, but a coherent molecular mechanism remains obscured by the lack of available structural data.

We have demonstrated that binding of a cargo adapter, nesprin-4, to the kinesin heterotetramer is sufficient to relieve one facet of the motor’s autoinhibition mechanism. However, a SKIP fragment containing KLC-binding motifs did not activate the motor in our reconstitution for unknown reasons (Figure 4). One clue may be related to the architecture and native cellular environment of the kinesin adapter. Nesprins are thought to act as trimers within the outer nuclear envelope (Sosa et al., 2013), which raises questions about the stoichiometry of interaction with the dimeric kinesin, as each nesprin-4 molecule contains only one W-acidic motif. On the other hand, SKIP contains tandem W-acidic motifs and associates with membranes peripherally (Pernigo et al., 2013). The oligomeric state of SKIP is unknown, and we suggest that the oligomeric state and the native environment could play key roles in the effectiveness of motor activation by kinesin cargo-adapter molecules. Similarly, JIP1 was insufficient to activate the motor in our assay, consistent with prior results in cell lysates (Blasius et al., 2007; Kawano et al., 2012). Another molecule, FEZ1, that binds directly to the KHC was required to activate the kinesin heterotetramer in cell lysates (Blasius et al., 2007; Kawano et al., 2012). Further work is necessary to uncover whether autoinhibition, oligomerization, and posttranslational modifications play key roles in controlling the activity of kinesin cargo adapters.

We found that the activated kinesin-nesprin complex is much less efficient at initiating movement along MTs compared with truncated, constitutively active kinesin. Cargo binding to the KLC can unlock motor autoinhibition, but additional factors are required for full activation. Inclusion of MAP7 in our reconstitution greatly enhanced the landing of both activated and inhibited kinesin molecules onto MTs and also prolonged their run lengths. MAP7 alone, however, does not activate the kinesin heterotetramer, revealing a synergistic relationship requiring cargo-adapter binding to the KLC to relieve inhibition of motor activity and a direct interaction with a non-motor MAP to facilitate recruitment to MTs. We note there is also strong genetic synergy between kinesin and MAP7 *in vivo* (Metzger et al., 2012).

There are parallels between our findings for kinesin activation and the cytoplasmic dynein regulatory scheme, whereby a cargo-adapter molecule is required to facilitate the formation of a complex between dynein and its activator dynactin (McKenney et al., 2014; Schlager et al., 2014). Dynactin acts as a non-motor MAP that directs the landing of the activated dynein-dynactin-cargo-adapter complex onto tyrosinated MTs (McKenney et al., 2014, 2016) and concentrates dynein at MT plus ends (Moughamian et al., 2013; Xiang and Qiu, 2020). In cells, MAP7 targets kinesin to subsets of MTs (Serra-Marques et al., 2020), and future work focused on determining the mechanisms that target MAP7 to MTs will shed light on how cells regulate the spatiotemporal activity of the kinesin motor. Our results have broad implications for understanding how cells control intracellular transport, a process that underlies a growing list of human diseases.

### Limitations of the study

One limitation of this study is that *in vitro* assays may not contain all physiologically relevant proteins involved in kinesin regulation or at their physiological concentrations. Further work will be necessary to examine the molecular effects described here at physiologically relevant protein concentrations. Our work makes predictions that require future efforts to devise and execute experiments *in vivo* to confirm our proposed model for kinesin activation.

## STAR★METHODS

### RESOURCE AVAILABILITY

#### Lead contact

Further information and requests for resources and reagents should be directed to and will be fulfilled by the lead contact, Richard J. McKenney (rjmckenney@ucdavis.edu).

#### Materials availability

All unique reagents generated in this study are available from the lead contact without restriction.

#### Data and code availability

Data reported in this paper will be shared by the lead contact upon request. This paper does not report original code. Any additional information required to reanalyze the data reported in this paper is available from the lead contact upon request.

### EXPERIMENTAL MODEL AND SUBJECT DETAILS

Sf9 (*Spodoptera frugiperda*) cells were maintained as a suspension culture in Sf-900II serum-free medium (SFM) (Thermo Fisher Scientific) at 27°C according to the manufacturers recommendations. Cells were shaken at ~150 rpm on a rotary shaker. The culture density was maintained between 1.0 × 10^6^ ~ 8.0 × 10^6^ cells/mL during routine passage. Infection of bacmid was performed at 1.0 × 10^6^ cells/mL. Protein expression was performed by infection of a culture at ~2.0 × 10^6^ cells/mL with baculovirus at a 1:100 volume:volume ratio. Cells were not authenticated. The sex of the cells is unknown.

BL21-CodonPlus (DE3)-RIPL (Agilent) *E. Coli* transformed with each plasmid were grown at 37°C in LB medium with shaking at ~ 200 rpm. Cells were not authenticated.

### METHOD DETAILS

#### Plasmids

A cDNA plasmid encoding human *KIF5A* (BC146670), *KIF5B* (BC126281), *KIF5C* (BC110287), *KLC1* (BC008881), *SKIP* (BC040441) were purchased from Transomics (Huntsville, AL, USA). As the purchased *KIF5C* cDNA lacked a DNA sequence encoding aa 1-238 of KIF5C protein, a DNA fragment encoding aa 1-238 were synthesized by gBlocks (Integrated DNA Technologies, Coralville, IA, USA) to complete the sequence. Mouse *JIP1* cDNA (NM_011162) is a gift from Toshiharu Suzuki (Hokkaido University, Japan). A human MAP7 plasmid was used as previously described (Monroy et al., 2018). A cDNA plasmid encoding human Nesprin-4 (NM_001297735, natural variant harboring Q165H) is a kind gift from Dan Starr (UC Davis). For preparing K420B, a DNA fragment encoding human KIF5B aa 1-420 was codon-optimized for *E. coli* and synthesized by gBlocks (Integrated DNA Technologies, Coralville, IA, USA).

Gibson assembly was used in the following constructs except as otherwise noted. For K420 series, KIF5A (aa 1-416), KIF5B codon optimized for E.coli (encoding aa 1-420 of human KIF5B as noted above), and KIF5C (aa 1-416) were cloned into pET28a vector with a C-terminal mScarlet-StrepII tag. Full-length KLC1, KLC1-CC (aa 1-200), KLC1-TPR (aa 201-500), cytosol domain of Nesprin-4 (aa 1-240), SKIP (amino acids 1-310) and full-length JIP1 were cloned into pET28a vector with N-terminal 6xHis-2xStrepII-sfGFP-2xPrescission protease site. The WD/AA mutation was introduced into pET28a-Nesprin-4 using primers containing desired mutation which are complementary to opposite strands of the cDNA and internal primers of pET28a. For full-length KIF5 dimers, full length KIF5A, KIF5B or KIF5C were cloned into pACEBac1 vector with a C-terminal mScarlet-StrepII tag. Using the pACEBac1-full-length KIF5B, KIF5B-ΔHinge(Δ505-610) was prepared using primers connecting C-terminus of KIF5B aa 1-504 with N-terminus of KIF5B aa 611-963 and internal primers of pACEBac1. KIF5B-IAK/AAA, H129A and E178A were prepared using primers harboring the desired mutations and internal primers of pACEBac1. For KIF5-KLC1 heterotetramer, KLC1 was first cloned into pIDS vector with N-terminal 6xHis-FLAG. pIDS-KLC1 vector was fused with either pACEBac1-KIF5A, KIF5B or KIF5C using Cre-Lox recombination. All constructs were verified by Sanger sequencing.

#### Antibodies

Anti-KIF5 (H2, #MAB1614) was purchased from sigma-aldrich.

#### Protein expression and purification

Bacterial expression and preparation of K420A, K420B, K420C, Nesprin-4, SKIP and JIP1 were performed as below. BL21-CodonPlus (DE3)-RIPL (Agilent) *E. Coli* were transformed with each plasmid and grown at 37°C in LB medium with Kanamycin until an optical density at 600 nm (OD_600_) reaches 0.4. The cultures were allowed to cool down to room temperature and induced by 0.2 mM isopropyl-β-D-thiogalactoside overnight at 18°C. Cells were harvested and resuspended in 25 mL of lysis buffer (50 mM HEPES-KOH, pH 7.5, 150 mM KCH3COO, 2 mM MgSO4, 1 mM EGTA, 10% glycerol) supplemented with 1 mM DTT, 1 mM PMSF, DNaseI and 0.1 mM ATP. Cells were lysed by passage through an Emulsiflex C3 high-pressure homogenizer (Avestin). Then the lysates were centrifuged at 15,000 × g for 20 min at 4°C. The resulting supernatant were subject to affinity chromatography described below.

For constructs cloned into pACEBac1 or pACEBac1/pIDS vector, DH10MultiBac (Geneva Biotech) were transformed to generate bacmid. To prepare baculovirus, 1 × 10^6^ cells of Sf9 cells were transferred to each well of a tissue-culture treated 6 well plate. After the cells attached to the bottom of the dishes, about ~5 μg of bacmid were transfected using 6 μL of Cellfectin II reagent (Thermo Fisher Scientific). 5 days after initial transfection, the culture media were collected and spun at 3,000 × g for 3 min to obtain the supernatant (P1). Next, 50 mL of Sf9 cells (2 × 10^6^ cells/mL) was infected with 50 μL of P1 and cultured for 5 days to obtain P2 viral supernatant. The resulting P2 were used for protein expression. For protein expression, 400 mL of Sf9 cells (2 × 10^6^ cells/mL) were infected with 4 mL of P2 virus and cultured for 65 h at 27°C. Cells were harvested and resuspended in 25 mL of lysis buffer (50 mM HEPES-KOH, pH 7.5, 150 mM KCH_3_COO, 2 mM MgSO4, 1 mM EGTA, 10% glycerol) along with 1 mM DTT, 1 mM PMSF, 0.1 mM ATP and 0.5% Triton X-100. After incubating on ice for 10 min, the lysates were centrifuged at 15,000 × g for 20 min at 4°C. The resulting supernatant were subject to affinity chromatography described below.

For affinity chromatography, the supernatants were pumped over a column of Streptactin XT resin (IBA) for ~1 h at 4°C. The columns were then washed with excess amount of lysis buffer to remove unbound material and the proteins were eluted in lysis buffer containing 100 mM D-biotin. Eluted proteins were further purified as described below.

K420A, K420B and K420C were further purified via anion exchange chromatography using a TSKgel SuperQ-5PW (Tosoh bioscience) 7.5 mm ID x 7.5 cm column equilibrated in HB buffer (35 mM PIPES-KOH pH 7.2, 1 mM MgSO4, 0.2 mM EGTA, 0.1 mM EDTA, pH 7.2). Bound proteins were eluted with a 45 mL of linear gradient of 0-1 M KCl in HB buffer. Fractions containing the proteins were combined and concentrated on amicon spin filters with a 50 kDa cutoff after addition of 0.1 mM ATP and 10% glycerol. Concentrated proteins were frozen in LiN_2_ and stored at −80°C.

KLC1, SKIP-1-310 and JIP1 were further purified via size exclusion chromatography using Superose 6 10/300 increase GL (Cytiva) column equilibrated in lysis buffer. KLC1-CC, KLC1-TPR, Nesprin-4 and Nesprin-4-WD/AA were purified via size exclusion chromatography using Superdex 200 10/300 GL (Cytiva) column equilibrated in lysis buffer. Fractions containing the proteins were combined and concentrated on amicon spin filters with a 50 kDa cutoff. Concentrated proteins were frozen in LiN_2_ and stored at −80°C.

KIF5A, KIFB, KIF5C, KIF5B-Δhinge, KIF5B-IAK/AAA, KIF5B-H129A, KIF5B-E178A, KIF5A-KLC1, KIF5B-KLC1, KIF5B-KLC1 were further purified via size exclusion chromatography using a BioSep SEC-s4000 (Phenomenex) particle size 5 μm, pore size 500Å, 7.8 mm ID x 600 mm column equilibrated in GF150 buffer (25 mM HEPES-KOH, 150 mM KCl, 2 mM MgCl_2_, pH 7.2). Fractions containing the proteins were combined and concentrated on amicon spin filters with a 50 kDa cutoff after addition of 0.1 mM ATP and 10% glycerol. Concentrated proteins were frozen in LiN_2_ and stored at −80°C. sfGFP-MAP7 protein was purified from insect cells as previously described (Monroy et al., 2018).

#### SEC-MALS (size exclusion chromatography coupled to multiangle light scattering)

The purified proteins were analyzed using BioSep SEC-s4000 (Phenomenex) particle size 5 μm, pore size 500Å, 7.8 mm ID x 300 mm column equilibrated in GF150 buffer (25 mM HEPES-KOH, 150 mM KCl, 2 mM MgCl_2_) plumbed into an HPLC (Agilent 1100). Molecular masses were analyzed by an inline SEC-MALs system (Wyatt Technology) which included a miniDAWN TREOS to measure light scattering and an Optilab T-rEX to measure refractive index. Molar mass was calculated using ASTRA v. 6 software (Wyatt Technology).

#### MT assembly

Porcine brain tubulin was isolated using the high-molarity PIPES procedure and then labelled with biotin NHS ester, Dylight-650 NHS ester, AZDye 647 NHS ester or Dylight-405 NHS ester as described previously (http://mitchison.hms.harvard.edu/files/mitchisonlab/files/labeling_tubulin_and_quantifying_labeling_stoichiometry.pdf). Pig brains were obtained from a local abattoir and used within ~4 h after death. MTs were prepared by mixing 60 μM unlabeled tubulin, 3 μM biotin-labeled tubulin and 3 μM Dylight-405-labeled tubulin for single molecule assays and 60 μM unlabeled tubulin and 3 μM Dylight-650-labeled tubulin for gliding assays. Mixed tubulin was incubated at 37°C with 10 mM GTP for 15 min. Then 20 μM taxol was added to stabilize the polymerized MT and additional incubation was performed at 37°C for 30 min. MTs were pelleted by centrifugation at 20,000 × g for 10 min over a 25% sucrose cushion and the pellet was resuspended in 50 μL BRB80 (80 mM PIPES-KOH pH 6.8, 1 mM MgCl2 and 1 mM EGTA) containing 10 μM taxol.

#### TIRF assays

Glass chambers were prepared by acid washing as previously described (Tan et al., 2017). Dylight-405 or AZDye647/biotin labeled MTs were flowed into streptavidin adsorbed flow chambers and allowed to adhere for 5–10 min. Unbound MTs were washed away using assay buffer (90 mM HEPES-KOH pH 7.6, 50 mM KCH_3_COO, 2 mM Mg(CH_3_COO)_2_, 1 mM EGTA, 10% glycerol, 0.1 mg/mL biotin–BSA, 0.2 mg/ml K-casein, 2 mg/mL BSA, 0.5% Pluronic F127, 2 mM ATP, and an oxygen scavenging system composed of PCA/PCD/Trolox). Purified motor protein was diluted to indicated concentrations in the assay buffer. Then, the solution was flowed into the glass chamber. Sequential images of 488 and/or 561 channel were taken at the frame rate of 1 fps (Figures 5A, 5B and 5F), 2 fps (Figures 1E, 2E and 4C) or 10 fps (Figures S1C, 1I,2H, 3D, 4D, S5C and S5D), respectively. For quantification, kymographs were made by ImageJ software. Unidirectional lines greater than 3 pixels along x axis (corresponding to approximately 310 nm) were counted as processive runs and the other signals were counted as non-processive/static binding. Run lengths and velocities were obtained by hand picking of the beginning and ending points of each run and then converted to the actual values by calculation based on pixel values of x axis (distance) and y axis (time). For velocity analysis, the data were fit to a Gaussian distribution using GraphPad Prism 9, and the best-fit values of mean and SD were shown. Landing rates were calculated by dividing the total number of processive and non-processive runs by the length of the MT, total time of the kymograph and the concentration of the motor. Landing rates of processive runs were similarly calculated using the number of processive runs instead of the total number of processive and non-processive runs. For assays with MAP7, the total number of processive molecules were manually counted from kymographs of MTs in the presence of MAP7 alone or MAP7 + nesprin-4. Assays using AMP-PNP were performed using 2 mM AMP-PNP instead of ATP in the above. Prior to taking images, chambers were incubated at room temperature for 10 min to allow proteins time to react with AMP-PNP. For gliding assays, motor proteins were diluted in lysis buffer at 0.5 μM and first flowed into empty chambers. After incubating the chamber at room temperature for 10 min, residual proteins were washed away with assay buffer. Then 650-labeled MTs diluted in assay buffer were flowed in. MT channel were taken at 2 fps. Kymographs of MTs were prepared by ImageJ software and velocities were determined based on the pixel values of x axis (distance) and y axis (time). Images were acquired using a Micromanager (Edelstein et al., 2010) software-controlled Nikon TE microscope (1.49×NA, 100× objective) equipped with a TIRF illuminator and Andor iXon CCD EM camera. Fitting and statistical tests were performed in GraphPad PRISM 8. For analyzing velocities, Gaussian fitting was used for histograms of velocities. Best-fit values of mean and SD were shown. Statistical tests used in each assay are described in the legend of each figure.

#### Pull-down assays

Pull-down assays were performed with purified Nesprin-4, Nesprin-4-WD/AA. Rat brain was homogenized in equal weight: volume of buffer (50 mM Hepes, 50 mM Pipes pH 7.0, 1 mM EDTA, 2 mM MgSO4) using a dounce homogenizer and flash frozen in LiN_2_ and stored at −80°C. The frozen lysate was thawed on ice and supplemented with 2 mM DTT, 2 mM PMSF and 0.1% Nonidet P-40, followed by centrifugation at 270,000 × g for 10 min at 4°C. The supernatant was mixed with 300 nM of the StrepII-tagged proteins and incubated for 30 min on ice and the protein complexes were recovered by Strep agarose beads. The beads were washed with wash buffer (90 mM HEPES-KOH pH 7.6, 50 mM KCH_3_COO, 2 mM Mg(CH_3_COO)_2_, 1 mM EGTA, 10% glycerol, 0.1% NP-40)for 5 times and eluted with 3 mM d-desthiobiotin in wash buffer. The eluates were analyzed by SDS-PAGE and western blotting.

#### Analysis of protein sequences

Protein sequences were aligned by Jalview 2 and Clustal W. Identities between protein sequences were calculated with the SMS2 tool (https://www.bioinformatics.org/sms2/ident_sim.html).

### QUANTIFICATION AND STATISTICAL ANALYSIS

Figure legends detail all quantification and statistical analyses, including statistical tests used and exact values of N or n. N represents the number of trials and n represents the number of data points (molecules or MTs). Velocities were assembled into frequency distribution plots followed by a Gaussian fitting. Run lengths were assessed with a two-tailed unpaired Mann-Whitney test (between two groups) or a Kruskal-Wallis test followed by Dunn’s multiple comparison test (among more than two groups). Landing rates were assessed with a two-tailed unpaired Student’s *t*-test (between two groups) or one-way ANOVA followed by Tukey’s multiple comparison test (among more than two groups). Fluorescence intensities of MTs were assessed with a two-tailed unpaired Student’s *t*-test (between two groups) or one-way ANOVA followed by Tukey’s multiple comparison test (among more than two groups). Statistical analyses were carried out using GraphPad Prism 9 for MacOSX. Differences were considered significant at *p < 0.05, **p < 0.01, ***p < 0.001 and ****p < 0.0001. For velocity, best-fit values of mean ± SD from a Gaussian fitting were shown. Mean ± SD were shown for landing rates. Median and interquartile range were shown for run lengths.

## Supplementary Material

1

## Figures and Tables

**Table:** KEY RESOURCES TABLE

REAGENT or RESOURCE	SOURCE	IDENTIFIER
Antibodies		
Mouse anti-KIF5 (H2)	Merck Millipore	MAB1614; RRID:AB_94284
DyLight 680 goat anti-mouse IgG	ThermoFisher Scientific	35518
Bacterial and virus strains		
BL21-CodonPlus (DE3)-RIPL competent cells	Agilent	230280
XL10-Gold competent cells	Agilent	200314
Stbl3 competent cells	ThermoFisher Scientific	C737303
PirHC competent cells	Geneva Biotech	N/A
DH10MultiBac competent cells	Geneva Biotech	N/A
Chemicals, peptides, and recombinant proteins		
Dylight 405 NHS Ester	ThermoFisher Scientific	46401
Dylight 650 NHS Ester	ThermoFisher Scientific	62265
AZDye 647 NHS ester	Fluoroprobes	1121-1
Biotin NHS ester		
Biotinylated poly(L-lysine)-[g]-poly(ethylene-glycol) (PLL-PEG-Biotin	SuSoS AG	PLL(20)-G[3.5]-PEG(2)/PEG(3.4)-biotin(50%)
Streptavidin	ThermoFisher Scientific	21135
Trolox (6-hydroxy-2,5,6,7,8-tetramethylchroman-2-carbonsaure, 97%)	Acros	AC218940050
3,4-Dihydroxybenzoic Acid (Protocatechuic acid)	Sigma-Aldrich	37580
Protocatechuate 3,4-Dioxygenase from Pseudomonas sp.	Sigma-Aldrich	P8279
κ-caesin from bovine milk	Sigma-Aldrich	C0406
Bovine Serum Albumin, Biotinylated	ThermoFisher Scientific	29130
Paclitaxel	Sigma-Aldrich	T7402
Pluronic F-157	Sigma-Aldrich	P2443
Glass cover slides (18x18-1.5)	ThermoFisher Scientific	12-541A
SuperFrost Microscope slides	ThermoFisher Scientific	12-550-143
Adenosine 5′-triphosphate disodium salt hydrate	Sigma-Aldrich	A2383
Guanosine 5′-triphosphate sodium salt hydrate	Sigma-Aldrich	G8877
Bovine Serum Albumin	Sigma-Aldrich	A2058
DNase I	NEB	M0303L
DTT (dithiothreitol)	ThermoFisher Scientific	R0862
PMSF (Phenylmethylsulfonyl fluoride)	ThermoFisher Scientific	36978
Streptactin XT Superflow resin	IBA	2-4010-025
D-Biotin	CHEM-IMPEX	00033
Cellfectin II Reagent	ThermoFisher Scientific	10362100
Sf-900 II SFM	ThermoFisher Scientific	10902104
Cre recombinase	NEB	M0298L
Experimental models: Cell lines		
Sf9 cells	ThermoFisher Scientific	11496015
Recombinant DNA		
Human KIF5A	Transomics	BC146670
Human KIF5B	Transomics	BC126281
Human KIF5C	Transomics	BC110287
Human KLC1	Transomics	BC008881
Human SKIP	Transomics	BC040441
Mouse JIP1	T. Suzuki	NM_011162
Human Nesprin-4	D. Starr	NM_001297735
Human KIF5B (aa 1-420) codon optimized for *E. coli*	Integrated DNA Technologies	N/A
pFastBac His-2xStrepII-TEV-sfGFP-HsMAP7-FLAG	Monroy et al. (2018)	MOM9
pET28a HsKIF5A_1-416-mScarlet-StrepII	This study	MOM779
pET28a HsKIF5B_1-420 (codon optimized for *E. coli*)-mScarlet-StrepII	This study	MOM932
pET28a HsKIF5C_1-416-mScarlet-StrepII	This study	MOM780
pET28a His-2xStrepII-sfGFP-2xPPS-HsKLC1	This study	MOM575
pET28a His-2xStrepII-sfGFP-2xPPS-HsKLC1_CC (1-200)	This study	MOM633
pET28a His-2xStrepII-sfGFP-2xPPS-HsKLC1_TPR (201-500)	This study	MOM635
pET28a His-2xStrepII-sfGFP-2xPPS-HsNesprin4_isoform-2_ΔC	This study	MOM896
pET28a His-2xStrepII-sfGFP-2xPPS-HsNesprin4_isoform-2_ΔC_WD/AA	This study	MOM988
pET28a 2xStrepII-sfGFP-2xPPS-HsSKIP_1-310	This study	MOM622
pET28a His-2xStrepII-sfGFP-2xPPS-MmJIP1	This study	MOM511
pACEBac1 HsKIF5A-2xPPS-mScarlet-StrepII	This study	MOM657
pACEBac1 HsKIF5B-2xPPS-mScarlet-StrepII	This study	MOM735
pACEBac1 HsKIF5C-2xPPS-mScarlet-StrepII	This study	MOM658
pACEBac1 HsKIF5B_D505-610-2xPPS-mScarlet-StrepII	This study	MOM738
pACEBac1 HsKIF5B_IAKAAA-2xPPS-mScarlet-StrepII	This study	MOM789
pACEBac1 HsKIF5B_H129A-2xPPS-mScarlet-StrepII	This study	MOM790
pACEBac1 HsKIF5B_E178A-2xPPS-mScarlet-StrepII	This study	MOM791
pIDS His-FLAG-HsKLC1	This study	MOM489
pACEBac1/pIDS HsKIF5A-2xPPS-mScarlet-StrepII His-FLAG-HsKLC1	This study	MOM659
pACEBac1/pIDS HsKIF5B-2xPPS-mScarlet-StrepII His-FLAG-HsKLC1	This study	MOM965
pACEBac1/pIDS HsKIF5C-2xPPS-mScarlet-StrepII His-FLAG-HsKLC1	This study	MOM661
Software and algorithms		
FIJI	*Nature Methods* 9, 676-682 (2012)	https://Fiji.sc/
GraphPad Prism	GraphPad Software	https://www.graphpad.com/scientific-software/prism/
SMS (Sequence Manipulation Suite)	*Biotechniques* 28,1102-1104 (2000)	https://www.bioinformatics.org/sms2/ident_sim.html
Jalview 2	*Bioinformatics* 25, 1189-119 (2009)	https://www.jalview.org
μManager	*Curr. Protoc. Mol. Biol*., Chapter 14 (2010) Unit14 20	https://micro-manager.org/
Clustal W	*Nucleic Acids Res*., 22, 4673-4680 (1994)	http://www.clustal.org/
ASTRA 6	Wyatt Technology	N/A
Other		
Superose 6 Increase 10/300 GL	Cytiva	29091596
Superdex 200 Increase 10/300 GL	Cytiva	28990944
TSKgel SuperQ-5PW 7.5 mm × 7.5 cm	Tosoh bioscience	0018257
BioSep 5 μm SEC-s4000 500 Å, LC Column 600 × 7.8 mm	Phenomenex	00K-2147-K0
BioSep 5 μm SEC-s4000 500 Å, LC Column 300 × 7.8 mm	Phenomenex	00H-2147-K0

**Table 1.** Measured motility parameters of tail-truncated motors , full-length KIF5 dimers, and KIF5-KLC1 heterotetramers

Summary of motility measurement of KIF5 isotypes						
	Microtubule gliding velocity	Single-molecule assay				
		Velocity (nm/s)	Landing rates	Motors/μm/s/μm	Run length (μm)	Processivity (%)
K420	KIF5A	ND	1,048 ± 173	162 ± 31	0.7 (0.55–0.93)	56
	KIF5B	ND	870 ±147	146 ± 26	0.69 (0.59–0.90)	49
	KIF5C	ND	887 ± 152	147 ± 35	0.62 (0.52–0.80)	53

KIF5	KIF5A	1,160 ± 66	1,182 ± 187	0.69 ± 0.20	1.00 (0.69–1.61)	70
	KIF5B	887 ± 26	1,002 ± 149	0.08 ± 0.03	1.00 (0.76–1.39)	31
	KIF5C	965 ± 27	1,001 ± 170	0.10 ± 0.04	0.76 (0.59–1.00)	27

KIF5-KLC1 heterotetramer	KIF5A	1,176 ± 47	1,051 ± 176	0.10 ± 0.04	1.49 (0.78–2.84)	41
	KIF5B	943 ± 33	ND	0.06 ± 0.04	ND	4
	KIF5C	944 ± 36	ND	0.06 ± 0.05	ND	0

Velocities (mean ± SD), landing rates (mean ± SD), run lengths (median and interquartile range), and processivity are shown. ND, not determined.

## Highlights

- Biophysical characterization of all human kinesin-1 (KIF5) isotypes
- Autoinhibition of KIF5 dimer mediated by a mechanism distinct from current models
- Repression of KIF5 activity by the KLC is relieved by binding of cargo adapters
- Cargo adapters and MAP7 act synergistically to relieve kinesin autoinhibition
