# Synergistic autoinhibition and activation mechanisms control kinesin-1 motor activity

**DOI:** 10.1016/j.celrep.2022.111016

**Published:** 2022-06-28

**Authors:** Kyoko Chiba, Kassandra M. Ori-McKenney, Shinsuke Niwa, Richard J. McKenney

In the originally published version of this paper, an incorrect definition for the term “MAP7” was introduced during the production process. The correct definition for MAP7 is “microtubule-associated protein 7,” which now appears with the article online.

The production team apologizes for the error.

