# Supplementary material for: Synergistic autoinhibition and activation mechanisms control kinesin-1 motor activity
Source: Cell Rep. Author manuscript; Available in PMC 2022 Aug 11. (PMC9365671; doi:10.1016/j.celrep.2022.110900)
Supplement: 1 [file NIHMS1812260-supplement-1.pdf]

Cell Reports, Volume *39*

Supplemental information

Synergistic autoinhibition and activation mechanisms control kinesin-1 motor activity

Kyoko Chiba, Kassandra M. Ori-McKenney, Shinsuke Niwa, and Richard J. McKenney

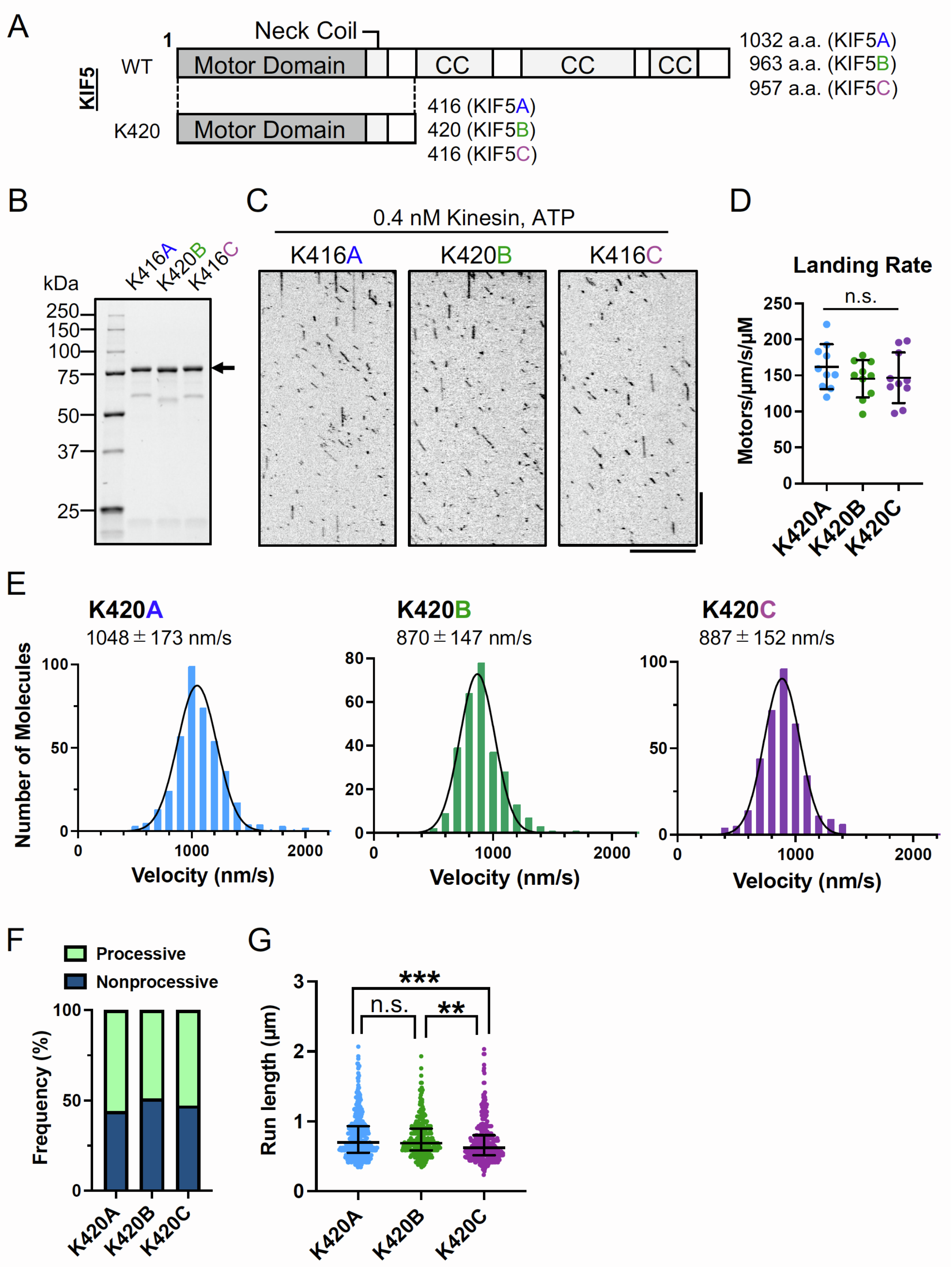

Figure S1. Characterization of tail-truncated, constitutively active KIF5 isotypes. Related to Figure 1.
(A) Schematic of full-length KIF5 and K420 constructs. Major domains are indicated. CC: coiled-coil. **(B)** Coomassie blue stained gel showing purified K420 proteins. Arrow indicates the full-length K420 proteins (74, 75 and 74 kDa for K420A, K420B and K420C, respectively). **(C)** Kymographs showing the motility of K420-mScarlet on MTs. Scale bars: 10 s (vertical) and 10 μm (horizontal). **(D)** Measured landing rates of K420s. Lines show mean ± SD: 162 ± 31 /μm/s/μM (K420A), 146 ± 26 /μm/s/μM (K420B) and 147 ± 35 /μm/s/μM (K420C). One-way ANOVA followed by Tukey's multiple comparison test was performed. n.s., not significant. N = 2. n = 10 MTs. **(E)** Histogram and Gaussian fit of motor velocities. mean ± SD: 1048 ± 173 nm/s (K420A), 870 ± 147 nm/s (K420B) and 887 ± 152 nm/s (K420C). n = 397, 282 and 359 molecules, respectively. **(F)** Measured frequency of processive and non-processive (static or diffusive) events. Processive runs are 56% (K420A), 49% (K420B) and 53% (K420C). n = 715, 573 and 679 molecules. **(G)** Scatter plots showing the run length of K420. Lines show median with interquartile: 0.70 (0.55 - 0.93) μm (K420A), 0.69 (0.59 - 0.90) μm (K420B) and 0.62 (0.52 - 0.80) μm (K420C). Kruskal-Wallis test followed by Dunn's multiple comparison test was performed. *** Adjusted $P < 0.001$; ** Adjusted $P < 0.01$; n.s., not significant. See also Table 1.

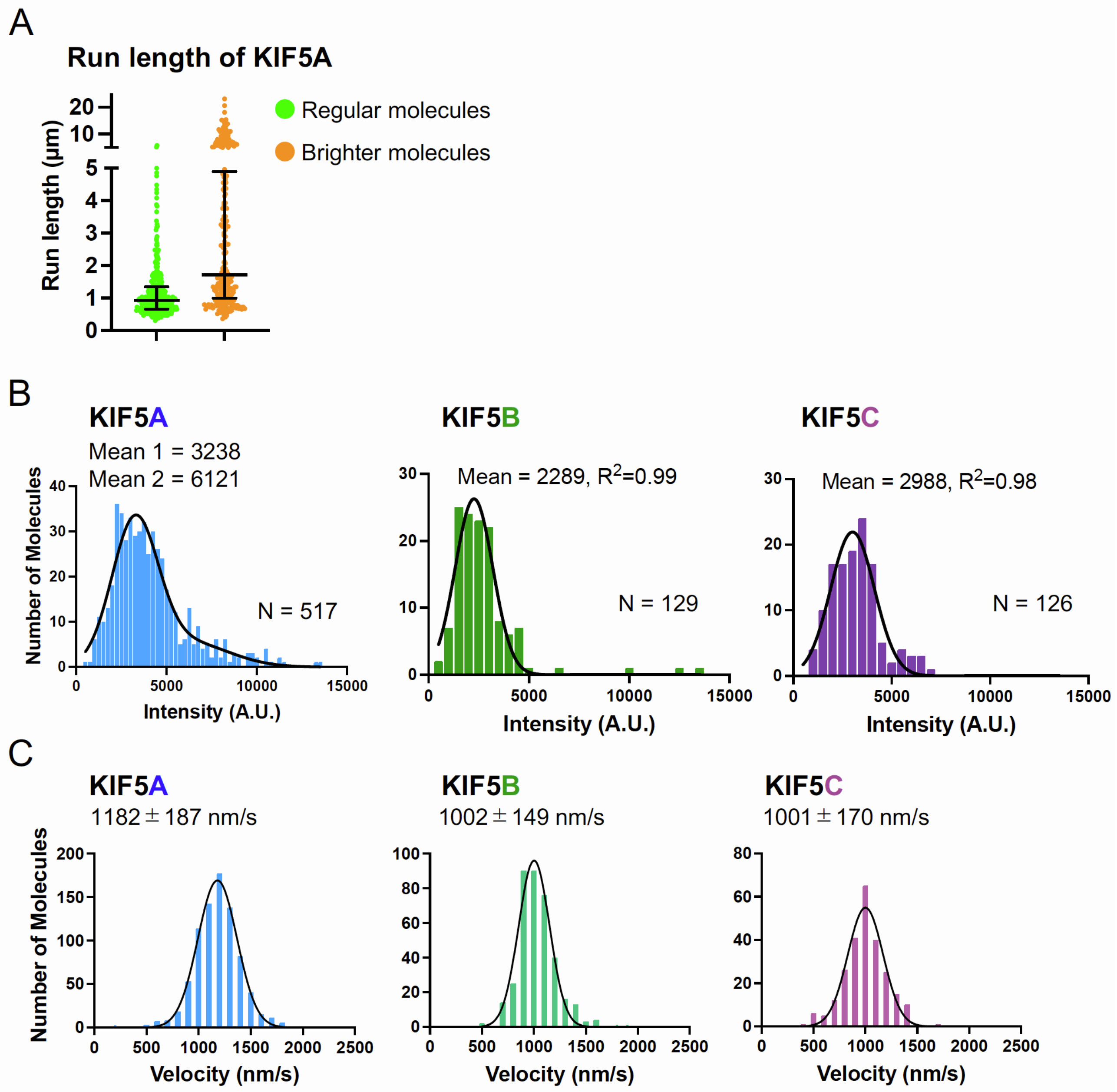

Figure S2. Further analysis of full length KIF5 proteins. Related to Figure 1.
(A) Measured run length of KIF5A classified by brightness of the molecules. Molecules that are 1.5 times brighter than regular molecules are classified as "Brighter". Lines show median with quartile: 0.93 (0.65 - 1.34) µm ("Regular") and 1.72 (1.00 – 4.90) µm ("Brighter"). A two-tailed unpaired Mann-Whitney test was performed. **** Adjusted P < 0.0001. N =1. n = 333, 236 molecules, respectively. **(B)** Histograms of the brightness of KIF5 observed in the single molecule assay. Intensities (A.U.) of each molecule subtracted background are plotted. The curves are sum of two Gaussians (KIF5A) or a Gaussian (KIF5B and KIF5C). Mean intensities (A.U.) based on the fitting are 3239 and 6127 (Mean 1 and Mean 2 of KIF5A), 2289 (KIF5B) and 2988 (KIF5C), respectively. N = 1. n = 517, 129 and 126 molecules for KIF5A, KIF5B and KIF5C, respectively. **(C)** Histogram and Gaussian fit of motor velocities of KIF5A, KIF5B and KIF5C measured on single molecule assay. The Gaussian fit are shown in Figure 1L. Velocity (mean ± S.D.): 1182 ± 187 nm/s (KIF5A), 1002 ± 149 nm/s (KIF5B) and 1001 ± 170 nm/s (KIF5C).

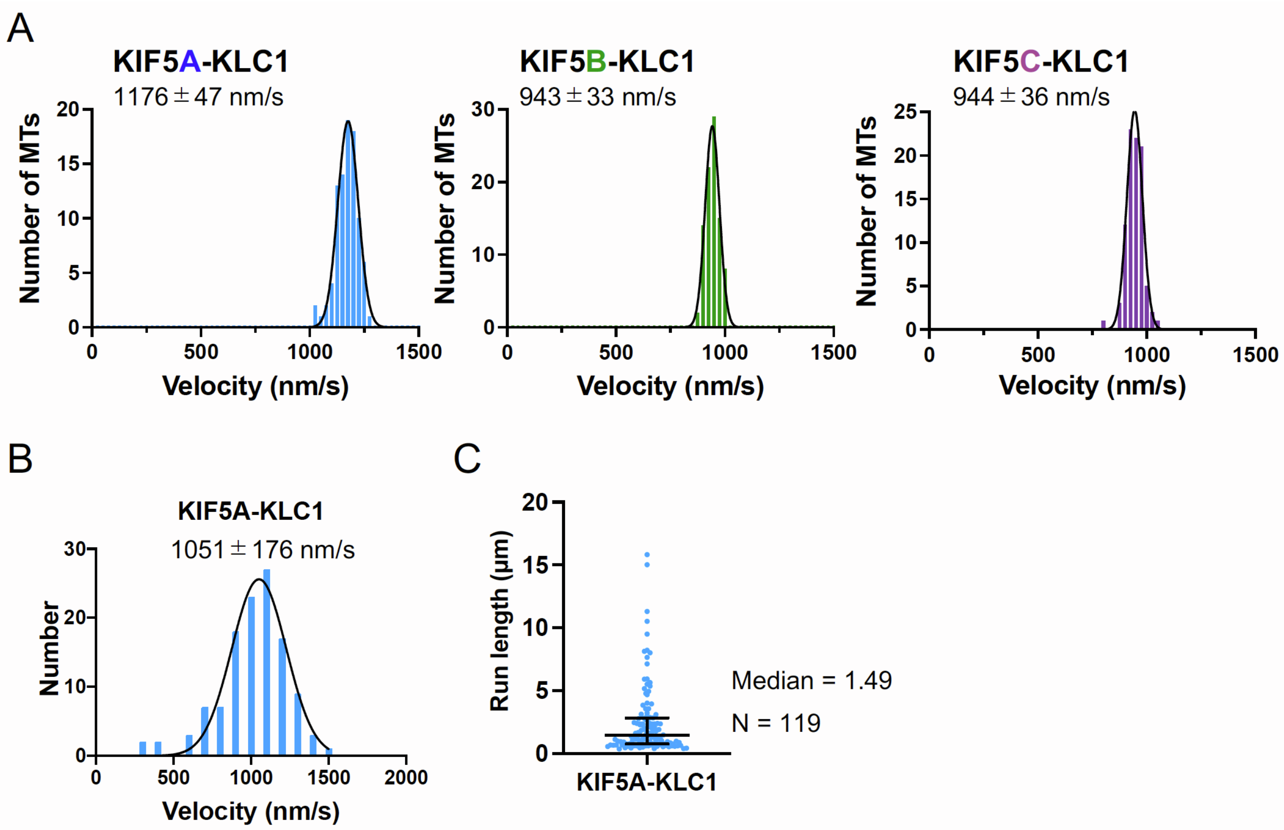

Figure S3. Further characterization of KIF5-KLC1 complexes. Related to Figure 2.
(A) Histogram and Gaussian fit of motor velocities of KIF5A-KLC1, KIF5B-KLC1 and KIF5C-KLC1 measured on MT gliding assay. The Gaussian fit are shown in Figure 2F. Velocity (mean ± SD): 1176 ± 47 nm/s (KIF5A-KLC1), 943 ± 33 nm/s (KIF5B-KLC1) and 944 ± 36 nm/s (KIF5C-KLC1), respectively. **(B)** Histogram and Gaussian fit of motor velocities of KIF5A-KLC1 measured on single molecule assay: 1051 ± 176 nm/s (mean ± SD). N = 2. n = 119 molecules. **(C)** Scatter plot showing the run length of KIF5A-KLC1. Lines show median with quartile: 1.49 (0.78-2.84) μm.

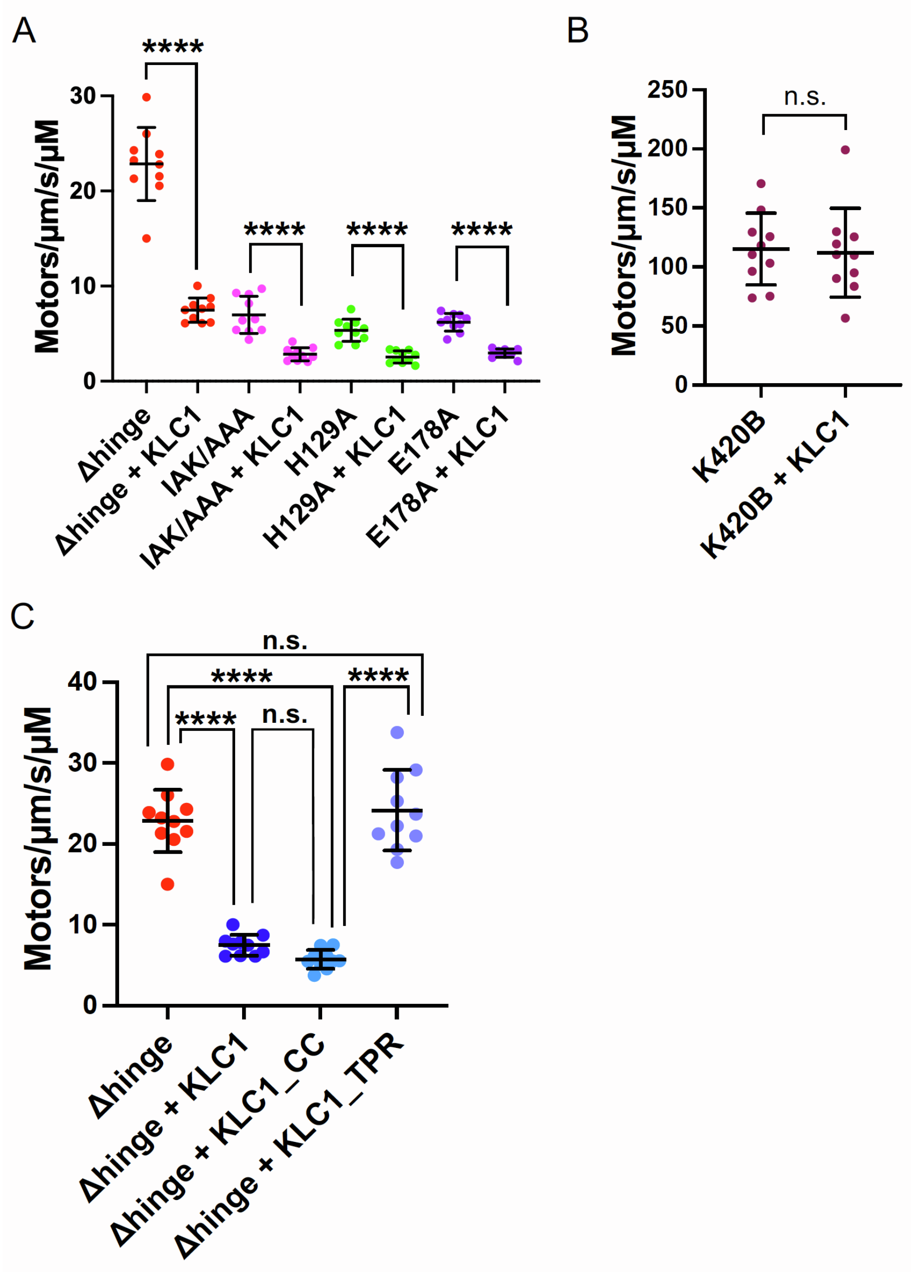

Figure S4. Further analysis of the effect of the KLC on KIF5 mutants. Related to Figure 3.
(A and B) Measured landing rates of KIF5B mutants or K420B in the presence or absence of full length KLC1. Motors landing events on MTs in the presence of ATP are counted based on kymographs (not shown). (A) 4 nM of KIF5B mutants with or without 20 nM KLC1. (B) 0.4 nM of K420B with or without 2 nM KLC1. Data for KIF5B-Δhinge, KIF5B-IAK/AAA, KIF5B-H129A and KIF5B-E178A are replotted from Fig. 3F. Lines show mean ± SD: 22.8 ± 3.8 motors/μm/s/μM (KIF5B-Δhinge), 7.5 ± 1.3 motors/μm/s/μM (KIF5B-Δhinge + KLC1), 7.0 ± 2.0 motors/μm/s/μM (KIF5B-IAK/AAA), 2.8 ± 0.7 motors/μm/s/μM (KIF5B-IAK/AAA + KLC1), 5.4 ± 1.2 motors/μm/s/μM (KIF5B-H129A), 2.6 ± 0.6 motors/μm/s/μM (KIF5B-H129A + KLC1), 6.2 ± 0.9 motors/μm/s/μM (KIF5B-E178A), 3.0 ± 0.4 motors/μm/s/μM (KIF5B-E178A + KLC1), 115 ± 30 motors/μm/s/μM (K420B), 112 ± 38 motors/μm/s/μM (K420B + KLC1). A two-tailed unpaired Student's *t*-test was performed. **** Adjusted $P < 0.0001$; n.s., not significant. N = 2, n = 10 MTs. **(C)** Measured landing rates of KIF5B-Δhinge in the presence or absence of full length KLC1, KLC1_CC or KLC1_TPR. KIF5's landing events on MTs in the presence of ATP are counted based on kymographs (not shown). 4 nM KIF5B-Δhinge with or without 20 nM KLC1. Lines show mean ± SD: 22.8 ± 3.8 motors/μm/s/μM (KIF5B-Δhinge), 7.5 ± 1.3 motors/μm/s/μM (KIF5B-Δhinge + KLC1), 5.7 ± 1.2 motors/μm/s/μM (KIF5B-Δhinge + KLC1_CC), 24.2 ± 5.0 motors/μm/s/μM (KIF5B-Δhinge + KLC1_TPR). Data for KIF5B-Δhinge alone and KIF5B-Δhinge + KLC1 are replotted from Fig. 3F and Fig S4A, respectively. One-way ANOVA followed by Tukey's multiple comparison test was performed. **** Adjusted $P < 0.0001$; n.s., not significant. N = 2, n = 10 MTs.

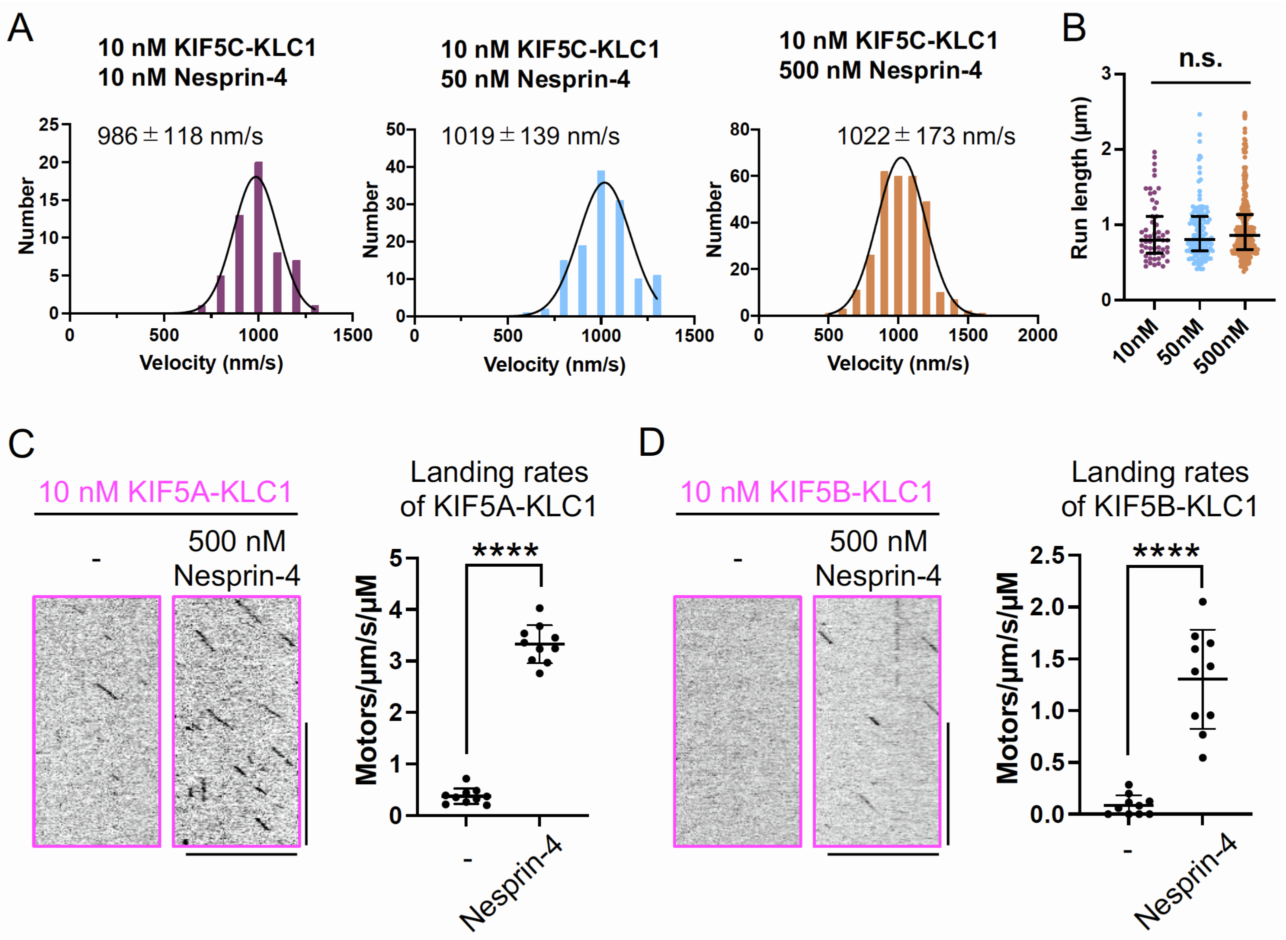

Figure S5. Further characterization of the effect of Nesprin-4 on KIF5-KLC1. Related to Figure 4.
(A) Histogram and Gaussian fit of motor velocities of KIF5C-KLC1 with 10 nM, 50 nM and 500 nM Nesprin-4. Velocities (mean ± SD): 986 ± 118 nm/s (10 nM KIF5C-KLC1 + 10 nM Nesprin-4), 1019 ± 139 nm/s (10 nM KIF5C-KLC1 + 50 nM Nesprin-4) and 1022 ± 173 nm/s (10 nM KIF5C-KLC1 + 500 nM Nesprin-4). N = 2. n = 56, 133 and 292 molecules. **(B)** Scatter plots showing the run length of KIF5C-KLC1 with 10 nM, 50 nM and 500 nM Nesprin-4. Lines show median with interquartile: 0.79 (0.62 - 1.11) µm (KIF5C-KLC1 + 10 nM Nesprin-4), 0.80 (0.65 - 1.11) µm (KIF5C-KLC1 + 50 nM Nesprin-4), 0.86 (0.67 - 1.14) µm (KIF5C-KLC1 + 500 nM Nesprin-4). Kruskal-Wallis test followed by Dunn's multiple comparison test was performed. n.s., not significant. **(C and D)** Representative kymographs showing the motility of 10 nM KIF5A-KLC1 or KIF5B-KLC1 with 500 nM Nesprin-4 on MTs. Scale bars: 10 s (vertical) and 10 µm (horizontal). Landing rates (mean ± SD): 0.37 ± 0.15 /µm/s/µM (10 nM KIF5A-KLC1), 3.33 ± 0.37 /µm/s/µM (10 nM KIF5A-KLC1 + 500 nM Nesprin-4), 0.09 ± 0.10 /µm/s/µM (10 nM KIF5B-KLC1), 1.30 ± 0.48 /µm/s/µM (10 nM KIF5B-KLC1 + 500 nM Nesprin-4). N = 2. n = 10 MTs, respectively. A two-tailed unpaired Student's *t*-test was performed. **** Adjusted P < 0.0001.
